# Supplementary material for: H3.1K27me1 loss confers Arabidopsis resistance to Geminivirus by sequestering DNA repair proteins onto host genome
Source: Nat Commun. 2023 Nov 18;14:7484. doi: 10.1038/s41467-023-43311-1 (PMC10657422; doi:10.1038/s41467-023-43311-1)
Supplement: Supplementary file 1 — Supplementary Information [file 41467_2023_43311_MOESM1_ESM.pdf]

Supplementary Information

Title: H3.1K27me1 loss confers Arabidopsis resistance to Geminivirus by sequestering DNA repair proteins onto host genome

Zhen Wang, Claudia M. Castillo Gonzalez, Changjiang Zhao, Chun-Yip Tong, Changhao Li, Songxiao Zhong, Zhiyang Liu, Kaili Xie, Jiaying Zhu, Zhongshou Wu, Xu Peng, Yannick Jacob, Scott D. Michaels, Steven E. Jacobsen, Xiuren Zhang*

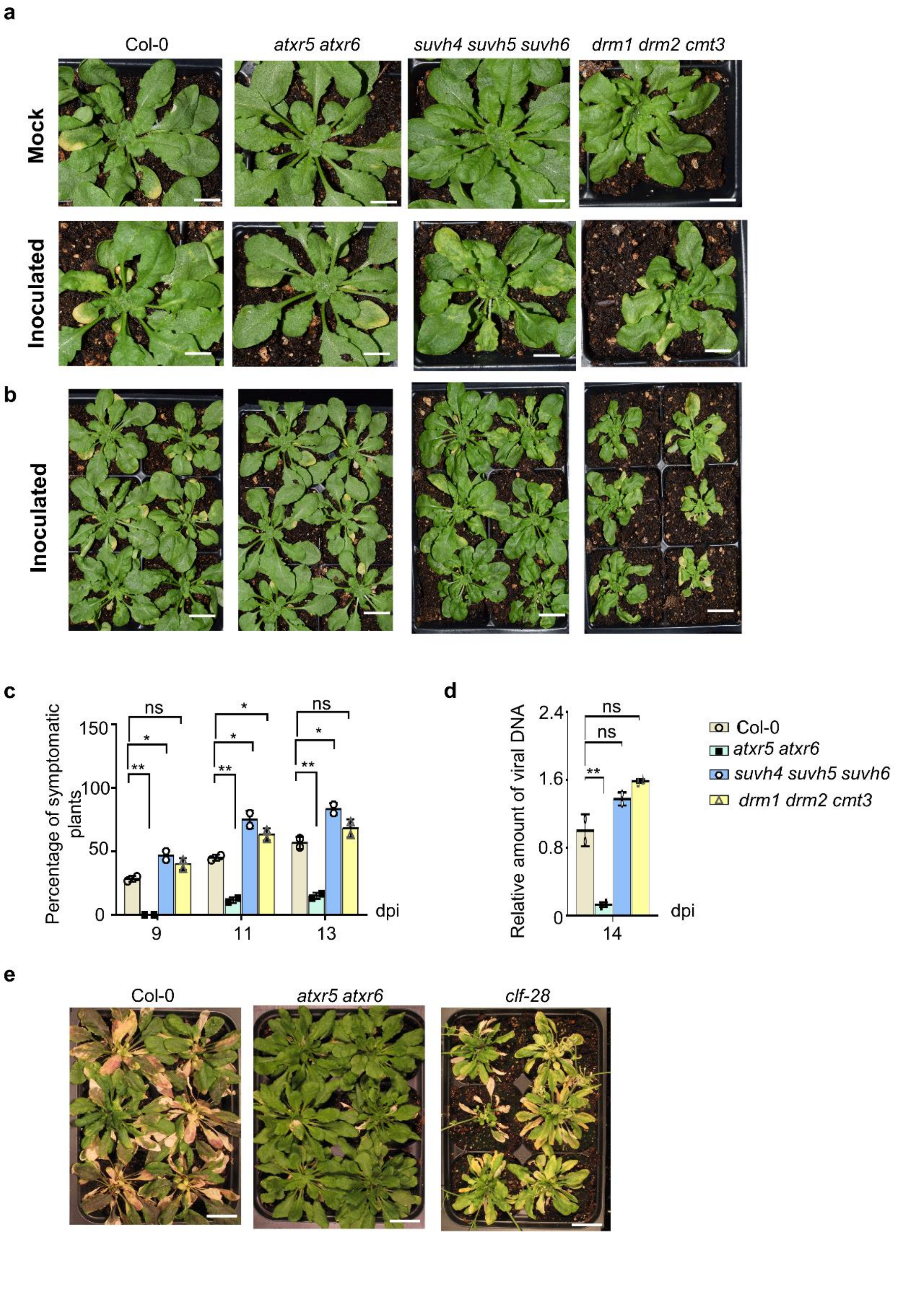

a
Col-0
atxr5 atxr6
suvh4 suvh5 suvh6
drm1 drm2 cmt3
Mock
Inoculated
b
Inoculated
c
Percentage of symptomatic plants
dpi
9
11
13
ns
*
**
d
Relative amount of viral DNA
dpi
14
ns
**
Col-0
atxr5 atxr6
suvh4 suvh5 suvh6
drm1 drm2 cmt3
e
Col-0
atxr5 atxr6
clf-28

Supplementary Fig. 1. Loss of function mutants of *ATXR5 and ATXR6*, different from other epigenetic mutants, display viral resistance phenotype. a, b, Representative pictures of the virus-inoculated plants in indicated backgrounds. Photographs were taken at 14 dpi. Scale bars, 1 cm (a). Scale bars, 2 cm (b). **c,** Percentages of symptomatic plants of virus-inoculated plants in indicated backgrounds at 9, 11, and 13 dpi. Each dot represents one biological replicate. The experiments were performed with 30 plants/replicate. Data are presented as mean ± SD (n = 2 biological replicates). **d,** q-PCR assays show the amount of viral DNA A in CaLCuV-inoculated plants indicated at 14 dpi. Data are presented as mean ± SD (n = 2 biological replicates). Normalization of viral DNA was conducted as **Fig. 2g. e,** Representative pictures of the virus-inoculated plants in indicated backgrounds. Photographs were taken at 21 dpi. Scale bars, 2 cm. Statistics in **Supplementary Fig. 1c,** and **1d** was performed with unpaired two-tailed student t-test, *, **, *** and ****, $P < 0.05$, 0.01, 0.001, and 0.0001, respectively. Source data are provided in the Source Data File.

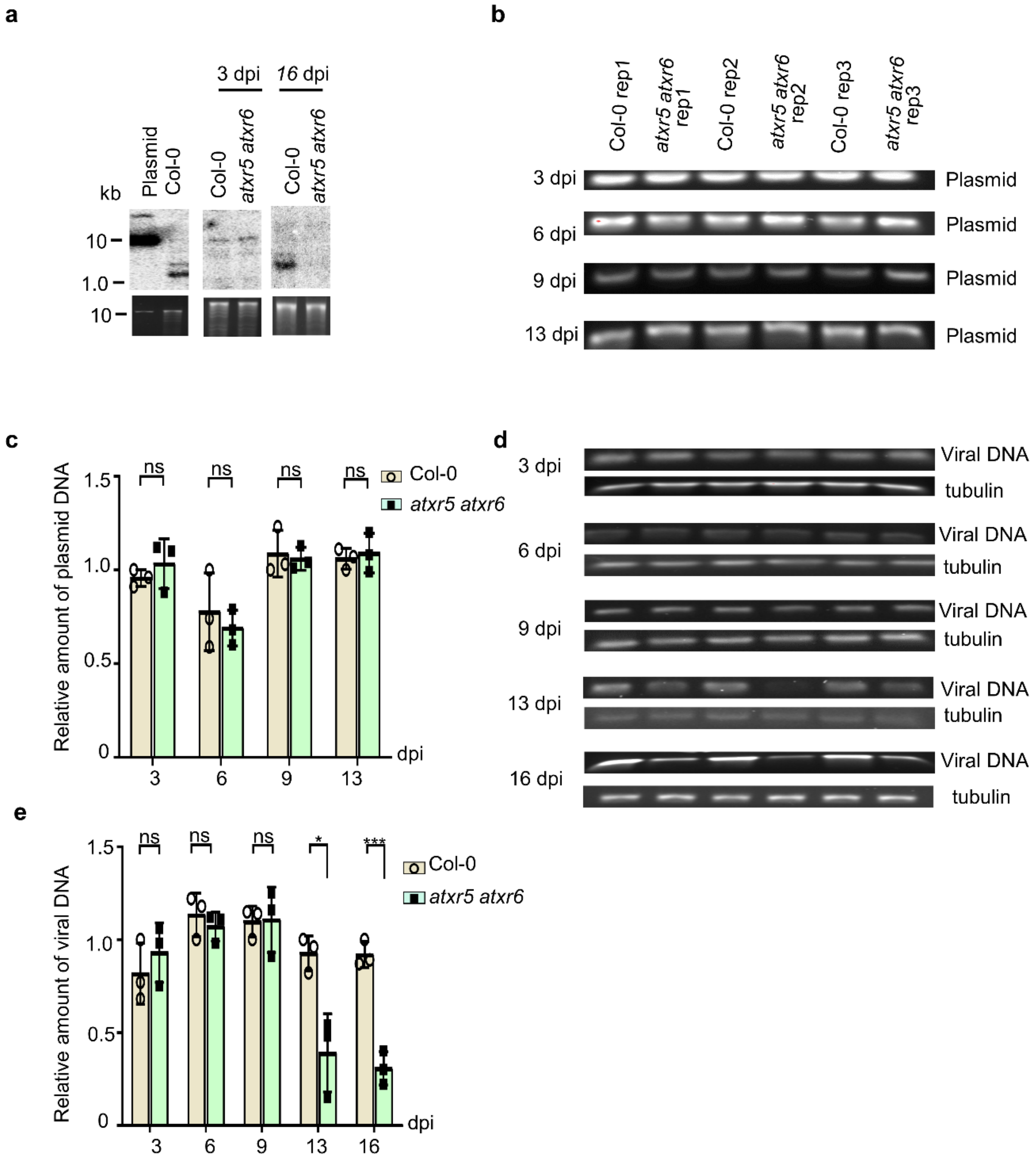

a
3 dpi
16 dpi
kb
Plasmid
Col-0
Col-0
atxr5 atxr6
Col-0
atxr5 atxr6
10
1.0
10
b
Col-0 rep1
atxr5 atxr6 rep1
Col-0 rep2
atxr5 atxr6 rep2
Col-0 rep3
atxr5 atxr6 rep3
3 dpi
6 dpi
9 dpi
13 dpi
Plasmid
c
Relative amount of plasmid DNA
ns
Col-0
atxr5 atxr6
1.5
1.0
0.5
0
3
6
9
13
dpi
d
3 dpi
6 dpi
9 dpi
13 dpi
16 dpi
Viral DNA
tubulin
e
Relative amount of viral DNA
ns
*
***
Col-0
atxr5 atxr6
1.5
1.0
0.5
0
3
6
9
13
16
dpi

Supplementary Fig. 2. Transfection efficiency is comparable between Col-0 and *atxr5 atxr6*. a, Southern blot assays showed the amount of delivered virus-contained plasmids (exemplified by 20 ng plasmid containing DNA A) and replicated viral DNA A (exemplified by 2 μg total DNA from virus-inoculated Col-0 in Fig. 1g) in CaLCuV-inoculated Col-0 and *atxr5 atxr6* at 3 and 16 dpi. EcoRI digested input DNA served as a loading control (Bottom panel). The experiments were repeated twice with similar results. **b,** Semi-quantitative PCR analysis to test the delivered plasmid levels in Col-0 and *axtr5 atxr6* at 3, 6, 9, and 13 dpi. *Tubulin* served as a control. The experiments were repeated two times with similar results. **c,** Quantitative-PCR assays to test virus plasmid level. The plasmid DNA detected by q-PCR was normalized against the *tubulin*, and then to Col-0 where the internally normalized viral titer of replicate 1 was arbitrarily assigned as a value of 1 at each time point. Each dot in the bar plot represents one replicate, experiments were performed with 6 plants/replicate. Data are presented as mean ± SD (n = 3 biological replicates). **d,** Semi-quantitative PCR analysis to test virus DNA levels in Col-0 and *axtr5 atxr6* at 3, 6, 9, and 13 dpi. *Tubulin* served as a control. The experiments were repeated twice with similar results. **e,** Quantitative-PCR assays to test virus DNA levels. Samples for **b, c, d,** and **e** come from the same set materials. Each dot in the bar plot represents one replicate, experiments were performed with 6 plants/replicate. Data are presented as mean ± SD (n = 3 biological replicates). Normalization was conducted according to **Supplementary Fig. 2c.** Statistics in **Supplementary Fig. 2c,** and **2e** were performed with unpaired two-tailed student t-test, *, **, *** and ****, $P < 0.05$, 0.01, 0.001, and 0.0001, respectively. Source data are provided in the Source Data File.

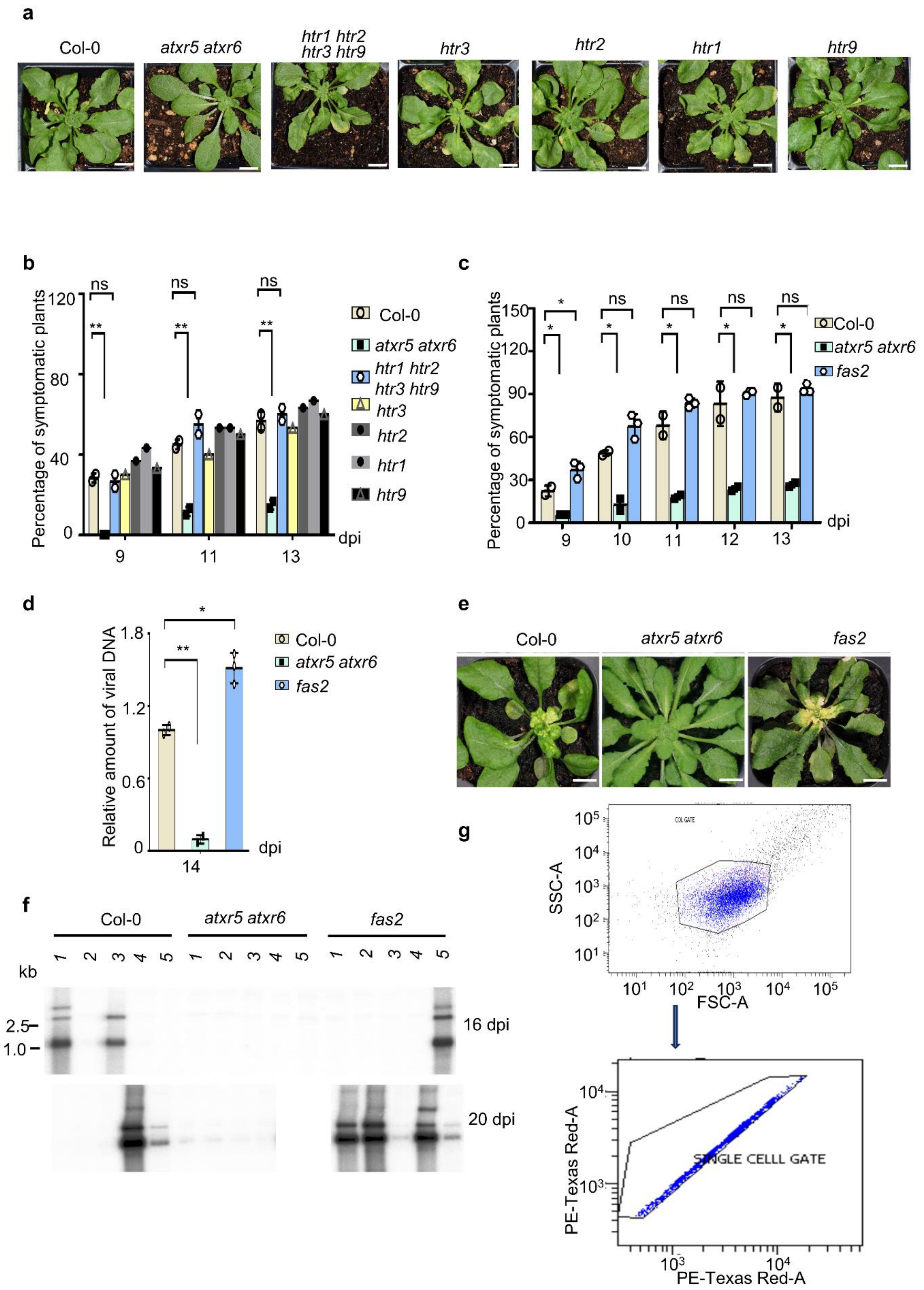
a
Col-0
atxr5 atxr6
htr1 htr2
htr3 htr9
htr3
htr2
htr1
htr9
b
Percentage of symptomatic plants
ns
**
120
80
40
0
9
11
13
dpi
Col-0
atxr5 atxr6
htr1 htr2
htr3 htr9
htr3
htr2
htr1
htr9
c
Percentage of symptomatic plants
*
ns
150
120
90
60
30
0
9
10
11
12
13
dpi
Col-0
atxr5 atxr6
fas2
d
Relative amount of viral DNA
*
**
1.8
1.2
0.6
0
14
dpi
Col-0
atxr5 atxr6
fas2
e
Col-0
atxr5 atxr6
fas2
f
Col-0
atxr5 atxr6
fas2
1 2 3 4 5
kb
2.5
1.0
16 dpi
20 dpi
g
COL GATE
SSC-A
FSC-A
PE-Texas Red-A
PE-Texas Red-A
SINGLE CELLL GATE

Supplementary Fig. 3. H3.1 single and quadruple mutants and *fas-2*, unlike *atxr5 atxr6*, are hyper-susceptible to viral infection. a, Representative pictures of the virus-inoculated Col-0, *atxr5 atxr6*, *htr1*, *htr2*, *htr3*, *htr9,* and *htr1 htr2 htr3 htr9* mutants. Photographs were taken at 14 dpi. Scale bars, 1 cm. **b,** Percentages of symptomatic plants of CaLCuV-inoculated Col-0, *atxr5 atxr6*, *htr1*, *htr2*, *htr3*, *htr9* and *htr1 htr2 htr3 htr9* mutants at 9, 11, and 13 dpi. Each dot in the bar plot represents one replicate, experiments were performed with 30 plants/replicate. Data are presented as mean ± SD (n = 2 biological replicates for Col-0, *atxr5 atxr6*, and *htr1 htr2 htr3 htr9*; n = 1 biological replicate for *htr3*, *htr2*, *htr1*, *htr9*). **c,** Percentages of symptomatic plants of CaLCuV-inoculated Col-0, *atxr5 atxr6* and *fas2* mutants at 9, 10, 11, 12, and 13 dpi. Each dot in the bar plot represents one replicate, experiments were performed with 36 plants/replicate. Data are presented as mean ± SD (n = 2 biological replicates for Col-0 and *atxr5 atxr6*; n = 3 biological replicate for *fas2*). **d,** Quantitative-PCR assays showed the amount of viral DNA A in CaLCuV-inoculated Col-0, and *fas2* at 14 dpi. Each dot in the bar plot represents one replicate, experiments were performed with 36 plants/replicate. Data are presented as mean ± SD (n = 2 biological replicates for Col-0 and *atxr5 atxr6*; n = 3 biological replicate for *fas2*). The relative amount of viral DNA A was normalized as described in **Fig. 2g**. **e,** Representative pictures of the virus-inoculated Col-0, *atxr5 atxr6*, and *fas2* at 21 dpi. Each dot in the bar plot represents one replicate, experiments were performed with three biological replicates (36 plants/replicate). Scale bars, 1cm. **f,** Southern blot analysis showed that the amount of viral DNA A in CaLCuV-inoculated individual plants of Col-0, *atxr5 atxr6,* and *fas2* mutants at 16 and 20 dpi. The experiments were repeated twice with similar results. **g.** The schematic diagram of experimental designs to collect sufficient intact protoplast (n=10000, Texas Red >400) to perform flow cytometry analysis. Statistics in **Supplementary Fig. 3b, 3c,** and **3d** were performed with unpaired two-tailed student t-test, *, **, *** and ****, $P < 0.05$, 0.01, 0.001, and 0.0001, respectively. The comparison between Col-0 and *fas2* in **Supplementary Fig. 3c,** and **3d** *was* performed with unpaired two-tailed student t-test with Welch's correction. Source data are provided in the Source Data File.

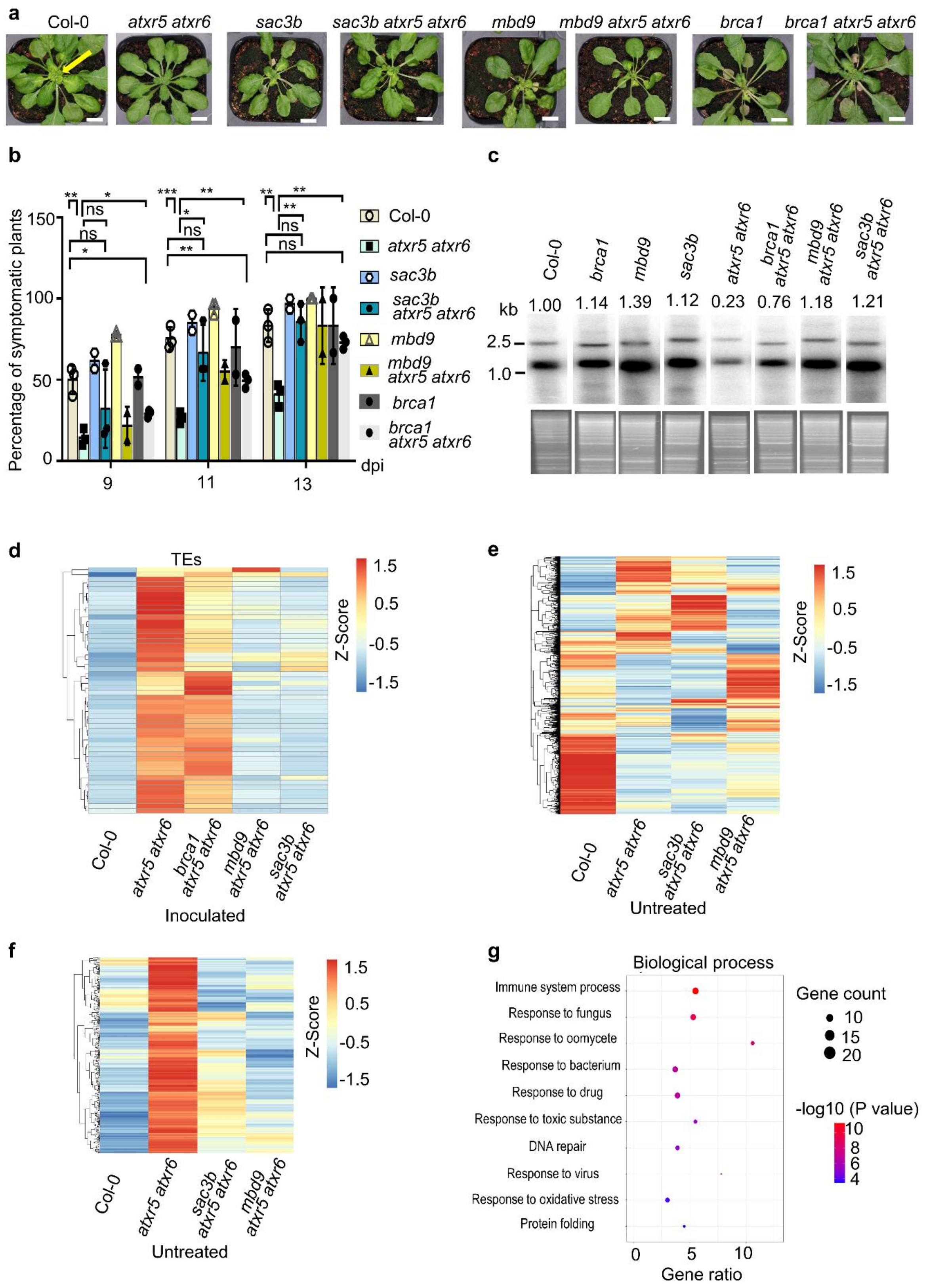

a
Col-0
atxr5 atxr6
sac3b
sac3b atxr5 atxr6
mbd9
mbd9 atxr5 atxr6
brca1
brca1 atxr5 atxr6
b
Percentage of symptomatic plants
dpi
9
11
13
ns
Col-0
atxr5 atxr6
sac3b
sac3b atxr5 atxr6
mbd9
mbd9 atxr5 atxr6
brca1
brca1 atxr5 atxr6
c
kb
2.5
1.0
1.00 1.14 1.39 1.12 0.23 0.76 1.18 1.21
d
TEs
Z-Score
Inoculated
e
Untreated
f
Untreated
g
Biological process
Immune system process
Response to fungus
Response to oomycete
Response to bacterium
Response to drug
Response to toxic substance
DNA repair
Response to virus
Response to oxidative stress
Protein folding
Gene ratio
Gene count
-log10 (P value)

Supplementary Fig. 4. The molecular phenotype of TE reactivation is uncoupled with the viral resistance phenotype in *atxr5 atxr6*. a, The loss of *MBD9*, *SAC3B*, or *BRCA1* could all increase viral susceptibility of *atxr5 atxr6*. Photographs of CaLCuV-inoculated plants were taken at 14 dpi. Scale bars, 1 cm. **b**, Percentages of symptomatic plants in different backgrounds induced by CaLCuV-inoculation at 9, 11, and 13 dpi. Each dot in the bar plot represents one replicate, experiments were performed with 30 plants/replicate. Data are presented as mean ± SD (n = 3 biological replicates for Col-0, *atxr5 atxr6*, *sac3b atxr5 atxr6, brca1 atxr5 atxr6,* and *mbd9;* n = 2 biological replicates for *brca1, sac3b,* and *mbd9 atxr5 atxr6*). c, Southern blot assays showed that the accumulation of viral DNA A in CalCuV-inoculated plants with different genotypes at 14 dpi. The experiments were repeated two times with similar results. EcoRI-digested input DNA serves as a loading control (Bottom panel). Scale bars, 2 cm. **d,** Heat map showed the change of transcript levels for TEs in virus-inoculated Col-0, *atxr5 atxr6, mbd9 atxr5 atxr6, brca1 atxr5 atxr6,* and *sac3b atxr5 atxr6*. The quantification was conducted by DESeq2. **e,** Heat map showed the overall change of transcript level for 1625 DEGs in *atxr5 atxr6, mbd9 atxr5 atxr6,* and *sac3b atxr5 atxr6.* RNA-seq data were mined from GSE77735. The quantification was done with DESeq2. **f,** Heat map showed the transcriptional level change for 240 genes selected from 1625 DEGs in indicated backgrounds. **g,** Bubble plots showed the enrichment of 240 DEGs in different GO biological. Statistics in **Supplementary Fig. 4b** were performed with unpaired two-tailed student t-test, *, **, *** and ****, $P < 0.05$, 0.01, 0.001, and 0.0001, respectively. Source data are provided in the Source Data File.

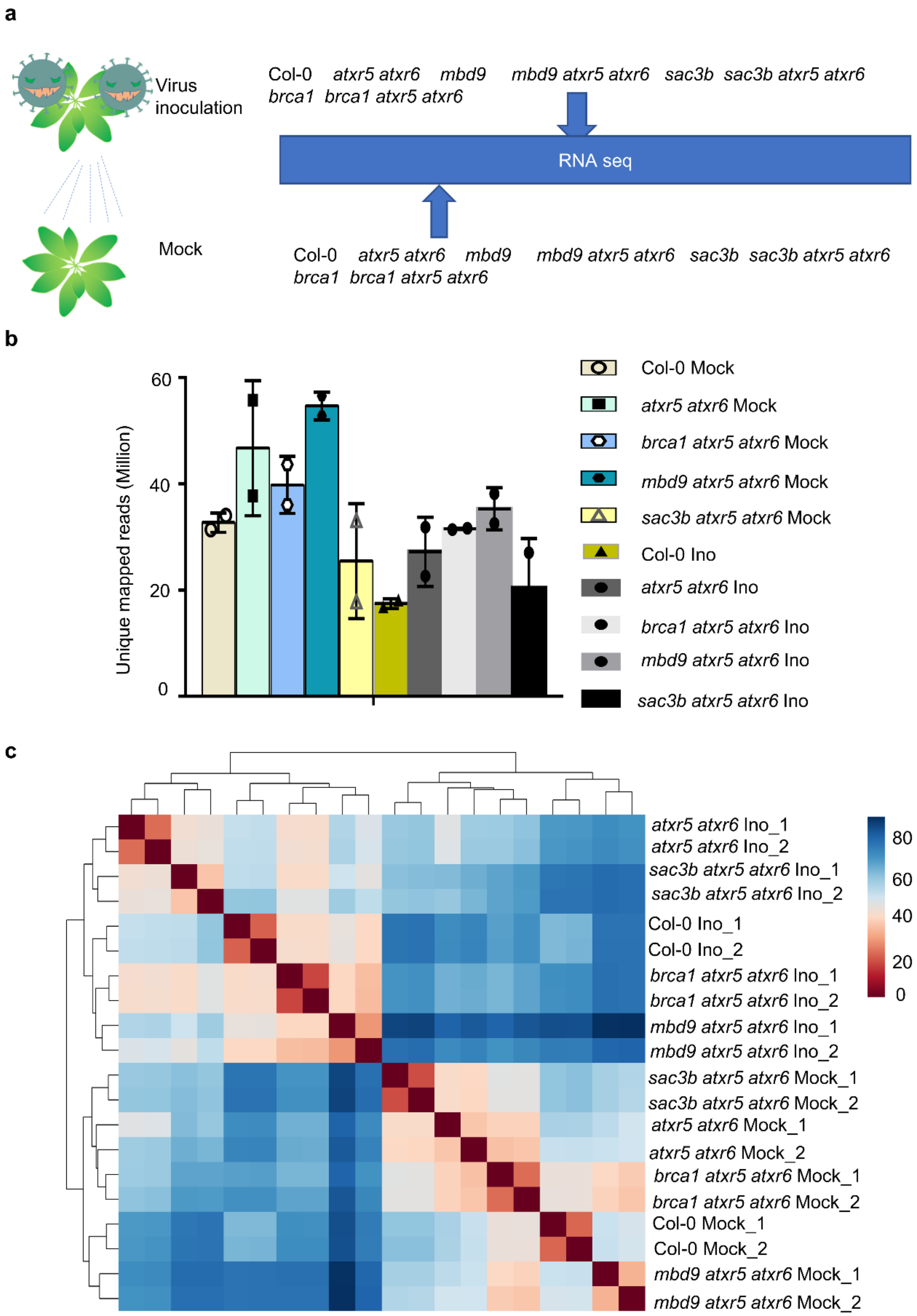
a
Virus inoculation
Mock
Col-0 atxr5 atxr6 mbd9 mbd9 atxr5 atxr6 sac3b sac3b atxr5 atxr6
brca1 brca1 atxr5 atxr6
RNA seq
Col-0 atxr5 atxr6 mbd9 mbd9 atxr5 atxr6 sac3b sac3b atxr5 atxr6
brca1 brca1 atxr5 atxr6
b
Unique mapped reads (Million)
60
40
20
0
Col-0 Mock
atxr5 atxr6 Mock
brca1 atxr5 atxr6 Mock
mbd9 atxr5 atxr6 Mock
sac3b atxr5 atxr6 Mock
Col-0 Ino
atxr5 atxr6 Ino
brca1 atxr5 atxr6 Ino
mbd9 atxr5 atxr6 Ino
sac3b atxr5 atxr6 Ino
c
atxr5 atxr6 Ino_1
atxr5 atxr6 Ino_2
sac3b atxr5 atxr6 Ino_1
sac3b atxr5 atxr6 Ino_2
Col-0 Ino_1
Col-0 Ino_2
brca1 atxr5 atxr6 Ino_1
brca1 atxr5 atxr6 Ino_2
mbd9 atxr5 atxr6 Ino_1
mbd9 atxr5 atxr6 Ino_2
sac3b atxr5 atxr6 Mock_1
sac3b atxr5 atxr6 Mock_2
atxr5 atxr6 Mock_1
atxr5 atxr6 Mock_2
brca1 atxr5 atxr6 Mock_1
brca1 atxr5 atxr6 Mock_2
Col-0 Mock_1
Col-0 Mock_2
mbd9 atxr5 atxr6 Mock_1
mbd9 atxr5 atxr6 Mock_2
80
60
40
20
0

Supplementary Fig. 5. Highly reproducible datasets show high quality of RNA-seq. a, The schematic diagram of experimental designs for viral inoculation and sample collection timing for RNA-seq. Each tray was separated into 6 parts and each part contained 6 individual plants. Figure is drawn with Microsoft Power Point. **b,** Summary of unique mapped reads used for following RNA-seq analysis. Ino represents viral-inoculated plants. **c,** Heatmap of sample clustering shows the high reproducibility within treatments for tested lines in RNA-seq.

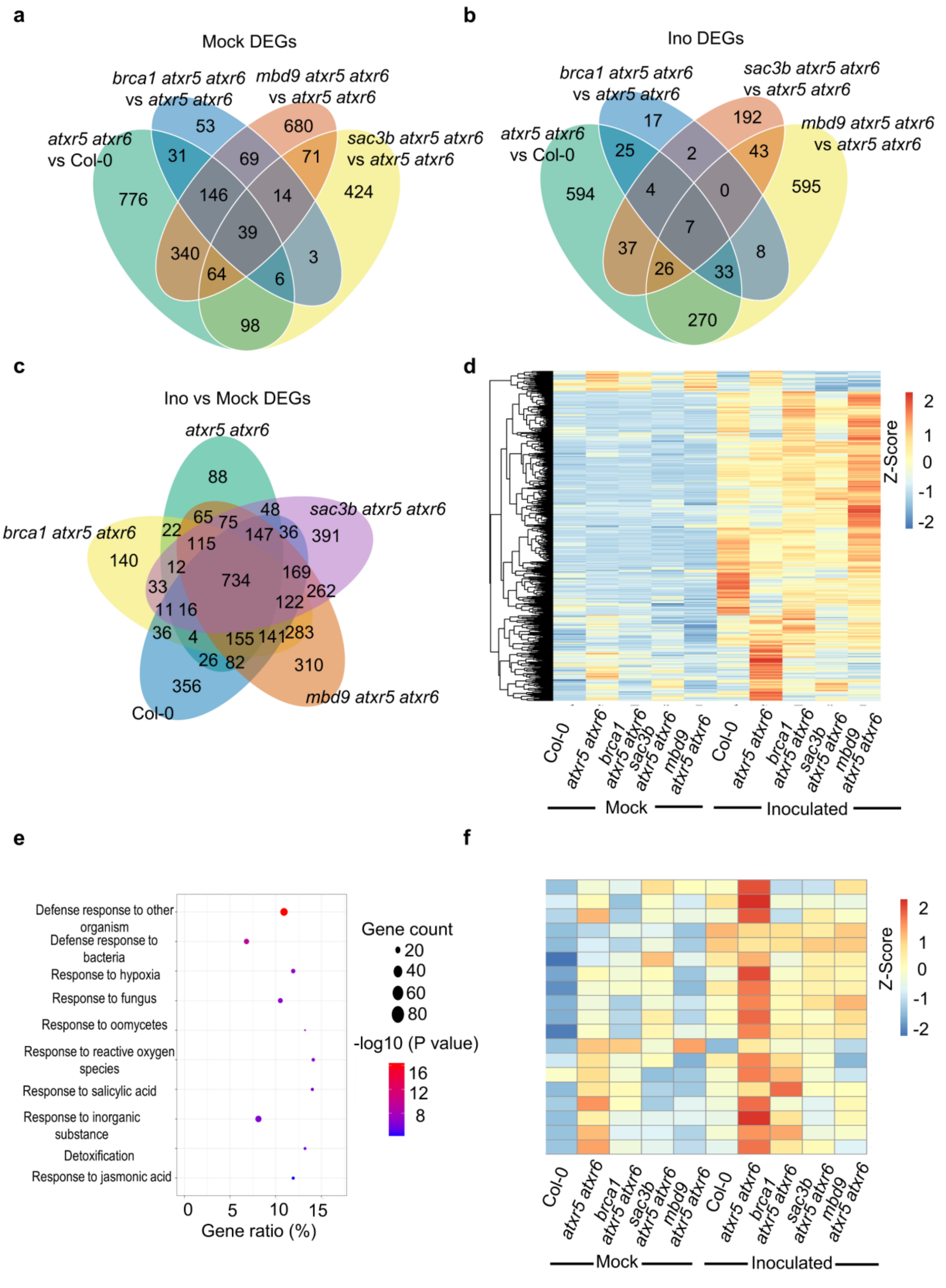
a
Mock DEGs
brca1 atxr5 atxr6 vs atxr5 atxr6
mbd9 atxr5 atxr6 vs atxr5 atxr6
atxr5 atxr6 vs Col-0
sac3b atxr5 atxr6 vs atxr5 atxr6
53
680
31
69
71
776
146
14
424
39
340
3
64
6
98
b
Ino DEGs
brca1 atxr5 atxr6 vs atxr5 atxr6
sac3b atxr5 atxr6 vs atxr5 atxr6
atxr5 atxr6 vs Col-0
mbd9 atxr5 atxr6 vs atxr5 atxr6
17
192
25
2
43
594
4
0
595
7
37
8
26
33
270
c
Ino vs Mock DEGs
atxr5 atxr6
88
sac3b atxr5 atxr6
brca1 atxr5 atxr6
mbd9 atxr5 atxr6
Col-0
48
65
75
22
147
36
391
115
140
12
169
734
33
262
122
11
16
36
4
155
141
283
26
82
310
356
d
Z-Score
2
1
0
-1
-2
Col-0
atxr5 atxr6
brca1 atxr5 atxr6
sac3b atxr5 atxr6
mbd9 atxr5 atxr6
Col-0
atxr5 atxr6
brca1 atxr5 atxr6
sac3b atxr5 atxr6
mbd9 atxr5 atxr6
Mock
Inoculated
e
Defense response to other organism
Defense response to bacteria
Response to hypoxia
Response to fungus
Response to oomycetes
Response to reactive oxygen species
Response to salicylic acid
Response to inorganic substance
Detoxification
Response to jasmonic acid
0
5
10
15
Gene ratio (%)
Gene count
20
40
60
80
-log10 (P value)
16
12
8
f
Z-Score
2
1
0
-1
-2
Col-0
atxr5 atxr6
brca1 atxr5 atxr6
sac3b atxr5 atxr6
mbd9 atxr5 atxr6
Col-0
atxr5 atxr6
brca1 atxr5 atxr6
sac3b atxr5 atxr6
mbd9 atxr5 atxr6
Mock
Inoculated

Supplementary Fig. 6. TWAS analysis identified the genes that are highly correlated with viral resistance of *atxr5 atxr6*. a, Venn diagram shows the overlap between DEGs from mock-treated samples. **b,** Venn diagram shows the overlap between DEGs from virus-inoculated samples. **c,** Venn diagram shows the DEGs between mock-treated and virus-inoculated samples. **d,** Heat map shows the changes of transcript levels for approximately 1100 selected genes from approximately 4800 differentially expressed genes from **a, b,** and **c** in mock-treated and virus-inoculated Col-0, *atxr5 atxr6*, *brca1 atxr5 atxr6*, *sac3b atxr5 atxr6,* and *mbd9 atxr5 atxr6* plants. **e,** Bubble plots show enrichment of DEGs from **d** in different GO biological processes. **f,** Heat map shows the changes of transcript levels for 19 genes related to DNA damage response (DDR) in mock-treated and virus-inoculated Col-0, *atxr5 atxr6*, *brca1 atxr5 atxr6*, *sac3b atxr5 atxr6,* and *mbd9 atxr5 atxr6* plants.

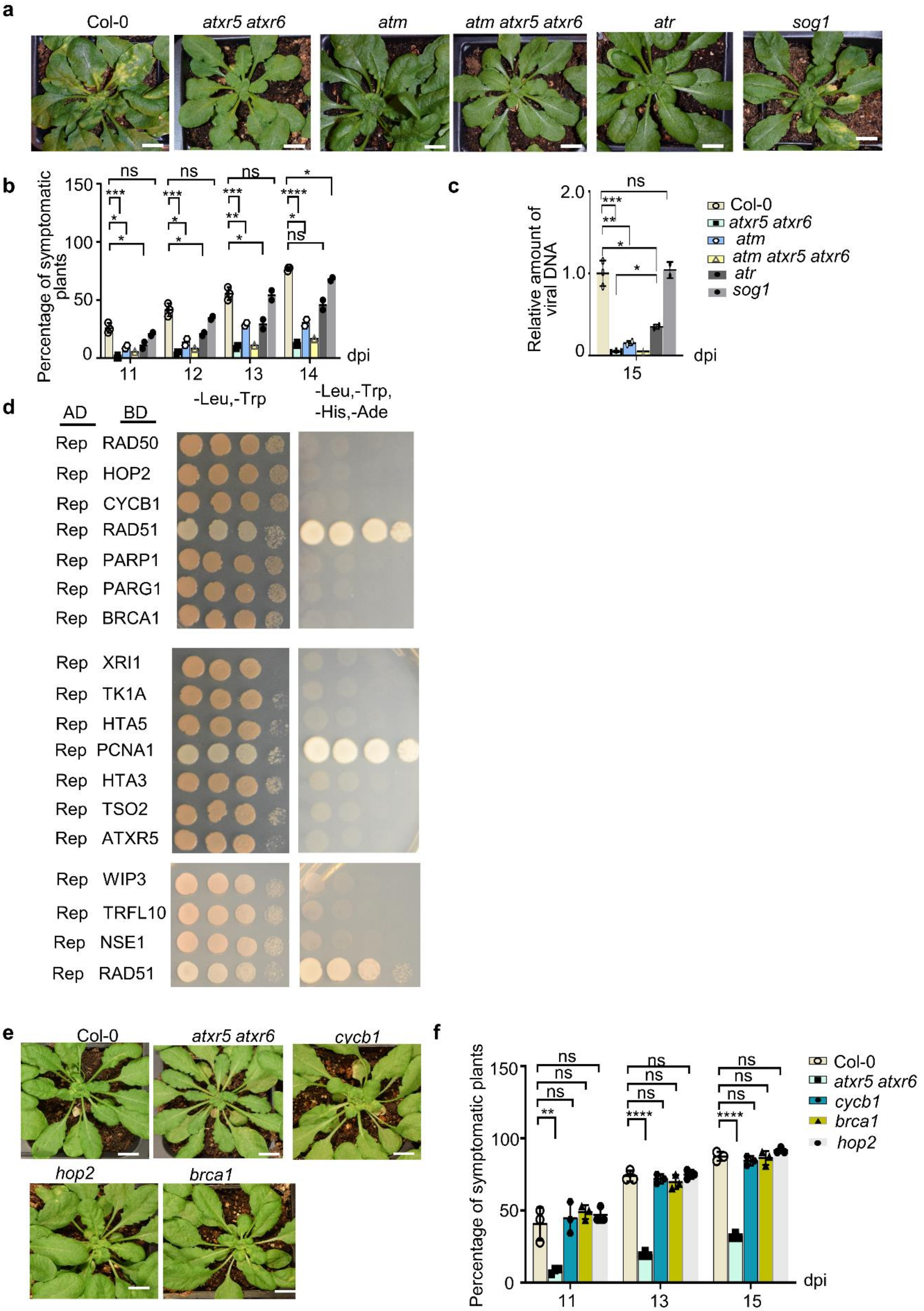
a
Col-0
atxr5 atxr6
atm
atm atxr5 atxr6
atr
sog1
b
Percentage of symptomatic plants
150
100
50
0
ns
***
*
*
****
dpi
11
12
13
14
c
Relative amount of viral DNA
2.0
1.0
0
ns
***
**
*
*
dpi
15
Col-0
atxr5 atxr6
atm
atm atxr5 atxr6
atr
sog1
d
AD
BD
-Leu,-Trp
-Leu,-Trp,
-His,-Ade
Rep RAD50
Rep HOP2
Rep CYCB1
Rep RAD51
Rep PARP1
Rep PARG1
Rep BRCA1
Rep XRI1
Rep TK1A
Rep HTA5
Rep PCNA1
Rep HTA3
Rep TSO2
Rep ATXR5
Rep WIP3
Rep TRFL10
Rep NSE1
Rep RAD51
e
Col-0
atxr5 atxr6
cycb1
hop2
brca1
f
Percentage of symptomatic plants
150
100
50
0
ns
**
****
dpi
11
13
15
Col-0
atxr5 atxr6
cycb1
brca1
hop2

Supplementary Fig. 7. Y2H screening of HRR proteins identify a few Rep-interacting proteins. a, Representative phenotypes of CaLCuV-inoculated Col-0, *atxr5 atxr6*, *atm*, *atxr5 atxr6 atm*, *atr,* and *sog1* plants. Photographs were taken at 15 dpi. Scale bars, 1 cm. **b,** Percentages of symptomatic plants induced by CaLCuV-inoculation in indicated backgrounds at 11, 12, 13, and 14 dpi. Each dot in the bar plot represents one replicate, experiments were performed with 32 plants/replicate. Data are presented as mean ± SD (n = 3 biological replicates for Col-0 and *atxr5 atxr6;* n = 2 biological replicates for *atm, atr,* and *sog1*; n = 1 biological replicate for *atxr5 atxr6 atm*). **c,** q-PCR shows the amount of viral DNA A in CaLCuV-inoculated plants in indicated backgrounds at 15 dpi. Each dot in the bar plot represents one replicate, experiments were performed with 32 plants/replicate. Data are presented as mean ± SD (n = 3 biological replicates for Col-0 and *atxr5 atxr6;* n = 2 biological replicates for *atm, atr* and *sog1*; n = 1 biological replicate for *atxr5 atxr6 atm*). The relative amount of viral DNA A was normalized as described in **Fig. 2g. d,** Y2H screening pinpointed the *bona fide* targets of Rep. **e,** Representative pictures of the virus-inoculated Col-0, *atxr5 atxr6*, *cycb1, hop2,* and *brca1* plants. Photographs were taken at 16 dpi. **f,** Percentages of symptomatic plants of CaLCuV-inoculated Col-0, *atxr5 atxr6*, *cycb1, hop2,* and *brca1* mutants at 11, 13, and 15 dpi. Each dot in the bar plot represents one replicate, experiments were performed with 32 plants/replicate. Data are presented as mean ± SD (n = 3 biological replicates). Statistics in **Supplementary Fig. 7b, 7c, and 7f** were performed with unpaired two-tailed student t-test, *, **, *** and ****, $P < 0.05$, 0.01, 0.001, and 0.0001, respectively. The comparisons between Col-0 and *atm, sog1,* or *atr* in **Supplementary Fig. 7b, 7c,** were performed with unpaired two-tailed student t-test with Welch's correction. Source data are provided in the Source Data File.

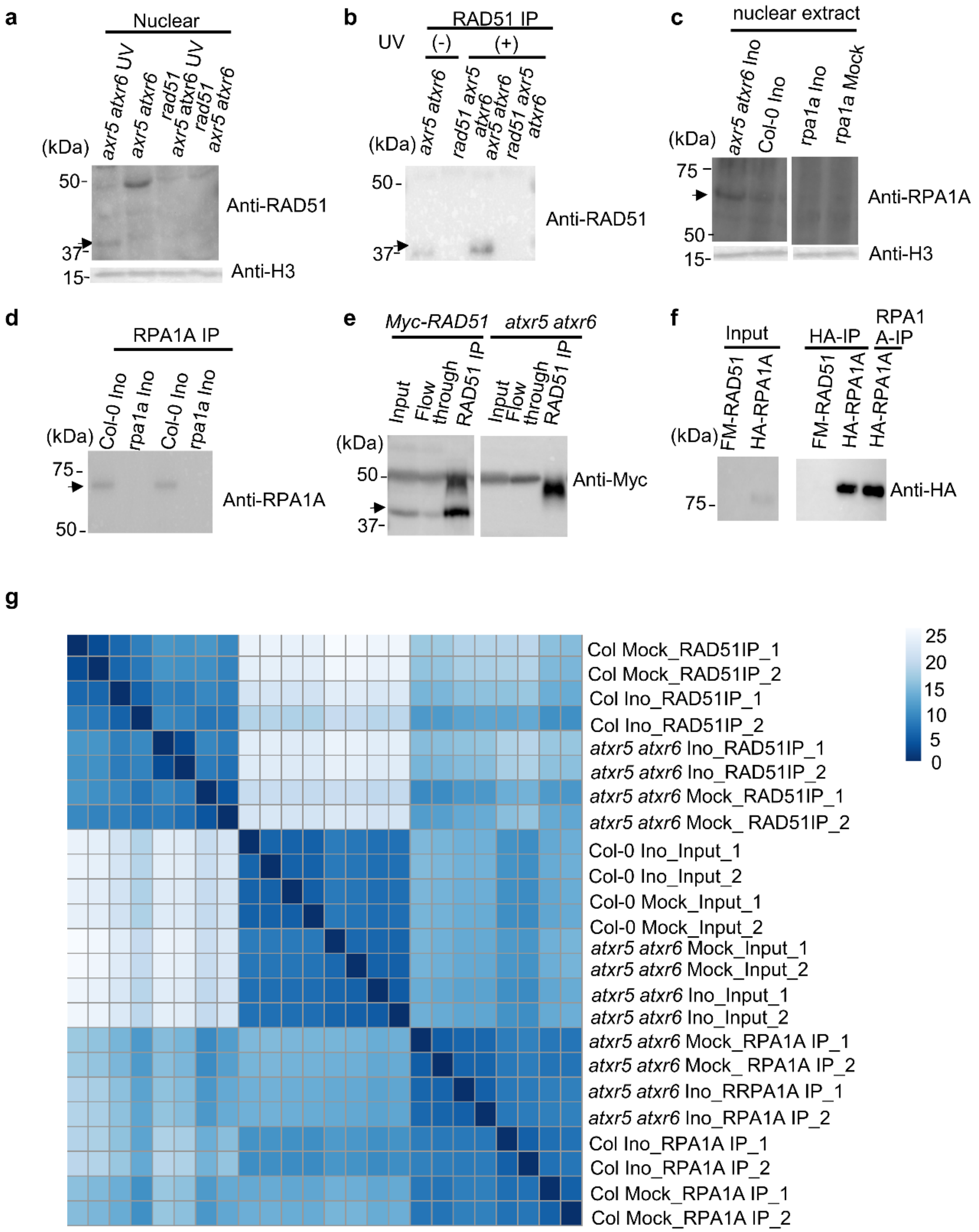
a
Nuclear
axr5 atxr6 UV
axr5 atxr6
rad51 axr5 atxr6 UV
rad51 axr5 atxr6
(kDa)
50
37
15
Anti-RAD51
Anti-H3
b
RAD51 IP
UV
(-)
(+)
axr5 atxr6
rad51 axr5 atxr6
axr5 atxr6
rad51 axr5 atxr6
(kDa)
50
37
Anti-RAD51
c
nuclear extract
axr5 atxr6 Ino
Col-0 Ino
rpa1a Ino
rpa1a Mock
(kDa)
75
50
15
Anti-RPA1A
Anti-H3
d
RPA1A IP
Col-0 Ino
rpa1a Ino
Col-0 Ino
rpa1a Ino
(kDa)
75
50
Anti-RPA1A
e
Myc-RAD51
atxr5 atxr6
Input
Flow through
RAD51 IP
Input
Flow through
RAD51 IP
(kDa)
50
37
Anti-Myc
f
Input
HA-IP
RPA1A-IP
FM-RAD51
HA-RPA1A
FM-RAD51
HA-RPA1A
HA-RPA1A
(kDa)
75
Anti-HA
g
Col Mock_RAD51IP_1
Col Mock_RAD51IP_2
Col Ino_RAD51IP_1
Col Ino_RAD51IP_2
atxr5 atxr6 Ino_RAD51IP_1
atxr5 atxr6 Ino_RAD51IP_2
atxr5 atxr6 Mock_RAD51IP_1
atxr5 atxr6 Mock_ RAD51IP_2
Col-0 Ino_Input_1
Col-0 Ino_Input_2
Col-0 Mock_Input_1
Col-0 Mock_Input_2
atxr5 atxr6 Mock_Input_1
atxr5 atxr6 Mock_Input_2
atxr5 atxr6 Ino_Input_1
atxr5 atxr6 Ino_Input_2
atxr5 atxr6 Mock_RPA1A IP_1
atxr5 atxr6 Mock_ RPA1A IP_2
atxr5 atxr6 Ino_RRPA1A IP_1
atxr5 atxr6 Ino_RPA1A IP_2
Col Ino_RPA1A IP_1
Col Ino_RPA1A IP_2
Col Mock_RPA1A IP_1
Col Mock_RPA1A IP_2
25
20
15
10
5
0

Supplementary Fig. 8. Quality analysis of ChIP-seq for RAD51 and RPA1A. a, c, Western blot analyses with isolated nuclei from plants with indicated genotypes and treatments using anti-RAD51 (a) and anti-RPA1A (c) antibodies. The experiments were repeated twice with similar results. **b, d,** Western blot analysis with immunoprecipitated RAD51 (b) and RPA1A (d) validated the specificity of anti-RAD51 and anti-RPA1A antibodies. The anti-RAD51 and anti-RPA1A antibodies were conjugated to protein A magnetics beads prior to immunoprecipitation. The experiments were repeated twice with similar results. **e,** Western blot analysis using an anti-Myc antibody validated the specificity of the anti-RAD51antibody used for immunoprecipitation. The IP of Myc-RAD51 is performed with an anti-RAD51 antibody in *Arabidopsis.* The experiments were repeated twice with similar results. **f,** Western blot analysis using an anti-HA antibody validated the specificity of the anti-RPA1A antibody for immunoprecipitation. The IP of HA-RPA1A is performed with anti-RPA1A in *N. benthamiana.* IP with anti-HA served as a positive control for IP with anti-RPA1A. The experiments were repeated twice with similar results. **g,** Heatmap of sample clustering shows high reproducibility between treatments for tested lines. Source data are provided in the Source Data File.

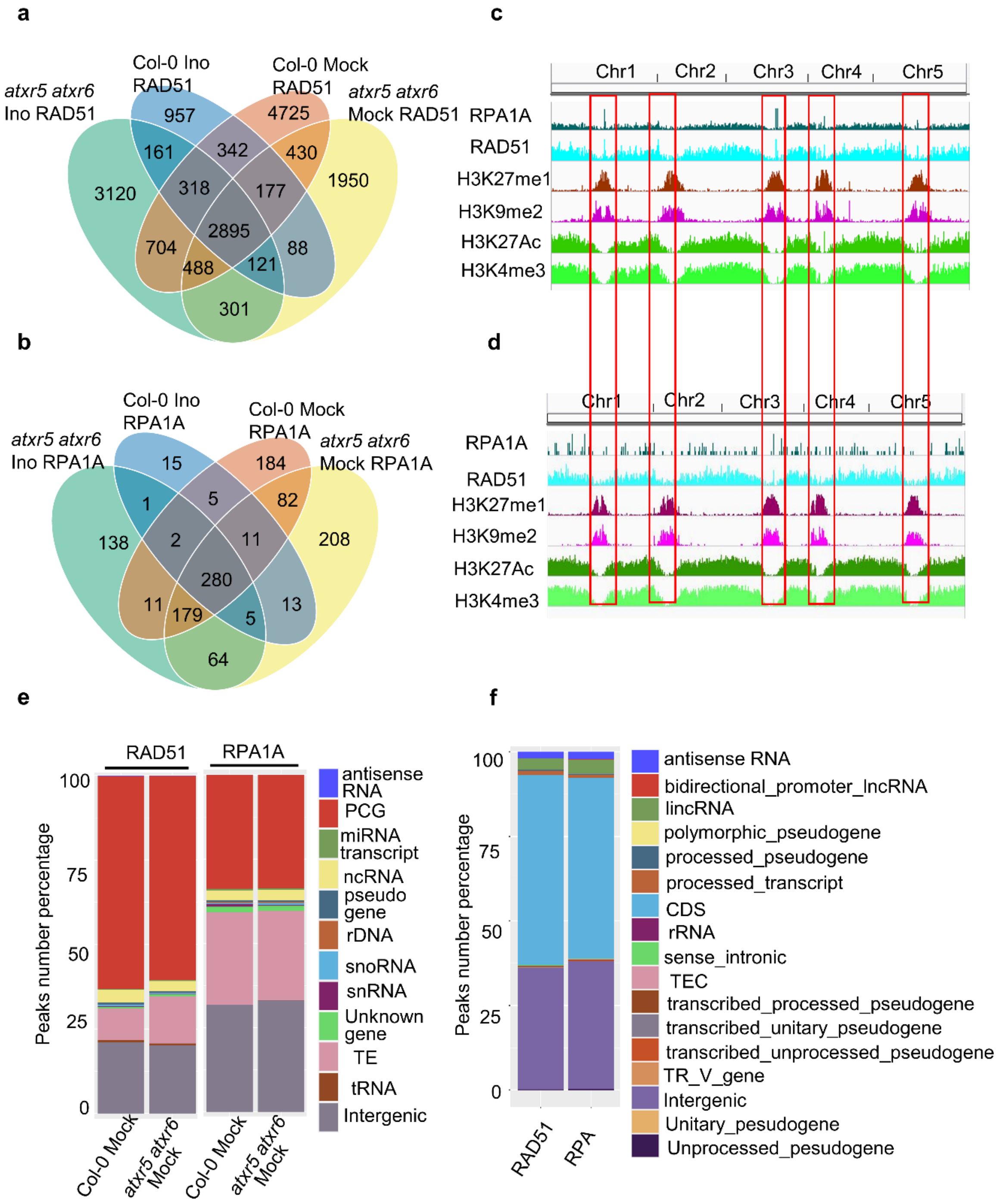
a
atxr5 atxr6 Ino RAD51
Col-0 Ino RAD51
Col-0 Mock RAD51
atxr5 atxr6 Mock RAD51
957
4725
161
342
430
3120
318
177
1950
2895
704
88
488
121
301
b
atxr5 atxr6 Ino RPA1A
Col-0 Ino RPA1A
Col-0 Mock RPA1A
atxr5 atxr6 Mock RPA1A
15
184
1
5
82
138
2
11
208
280
11
179
5
13
64
c
Chr1
Chr2
Chr3
Chr4
Chr5
RPA1A
RAD51
H3K27me1
H3K9me2
H3K27Ac
H3K4me3
d
Chr1
Chr2
Chr3
Chr4
Chr5
RPA1A
RAD51
H3K27me1
H3K9me2
H3K27Ac
H3K4me3
e
RAD51
RPA1A
Peaks number percentage
100
75
50
25
0
Col-0 Mock
atxr5 atxr6 Mock
Col-0 Mock
atxr5 atxr6 Mock
antisense RNA
PCG
miRNA transcript
ncRNA
pseudo gene
rDNA
snoRNA
snRNA
Unknown gene
TE
tRNA
Intergenic
f
Peaks number percentage
100
75
50
25
0
RAD51
RPA
antisense RNA
bidirectional_promoter_lncRNA
lincRNA
polymorphic_pseudogene
processed_pseudogene
processed_transcript
CDS
rRNA
sense_intronic
TEC
transcribed_processed_pseudogene
transcribed_unitary_pseudogene
transcribed_unprocessed_pseudogene
TR_V_gene
Intergenic
Unitary_pesudogene
Unprocessed_pesudogene

Supplementary Fig. 9. Distributions of RAD51-bound peak over euchromatin and heterochromatin regions. a, b Venn diagram shows the overlap of RAD51 (a), and RPA1A (b)-bound peaks among different genotypes and treatments. **c,** Chart diagrams show peak distributions of RPA1A, RAD51, H3K27me1, H3K9me2, H3K4me3, and H3K27Ac over genome of Col-0. Be noted: total mapping reads which contained multiple mapping reads were used to perform peak calling analysis (see methods). H3K9me2 and H3K27me1 related data were mined from published data (GSE111814), H3K4me3 related data were mined from published data (GSE166897), H3K27Ac related data were mined from GSE146126. **d,** Chart diagrams show the peak distributions of RPA1A, RAD51, H3K27me1, H3K9me2, H3K4me3, and H3K27Ac over genome of Col-0. Be noted that the unique mapping reads which does not contain multiple mapping reads are used to perform peak calling analysis. **e,** Genomic feature classification of RAD51 and RPA enriched regions in Col-0 and *atxr5 atxr6*. The y axis represents the percentages of peak numbers belonging to various categories. Peak identification using total mapped reads. **f,** Genomic feature classification of RAD51 and RPA enriched regions over genome of *Mus musculus*. The y axis represents the percentages of peak numbers belonging to various categories. Peak identification using total mapped reads. RAD51 and RPA related data were mined from published data (GSE143582). Source data are provided in the Source Data File.

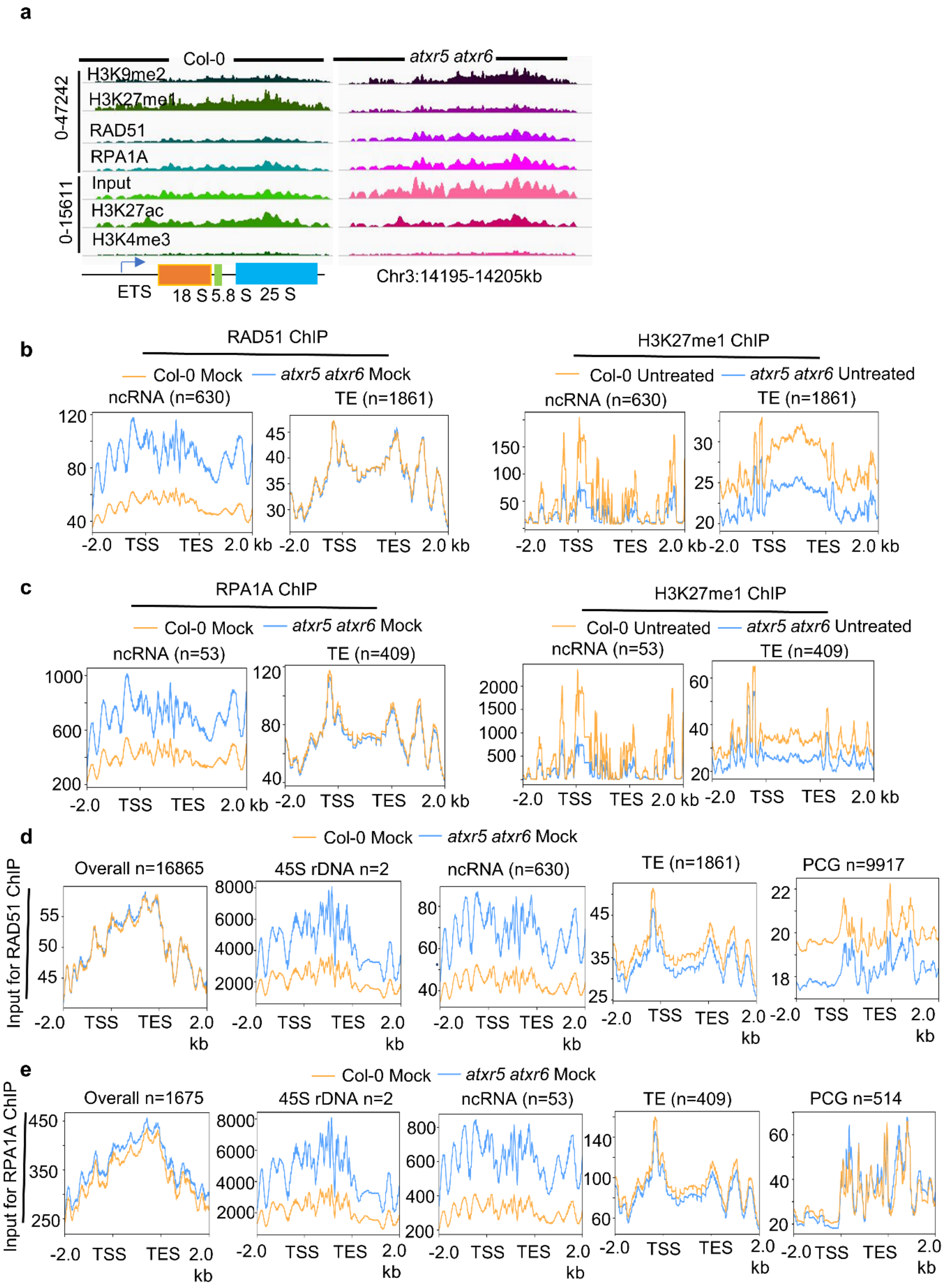

a
Col-0
atxr5 atxr6
0-47242
0-15611
H3K9me2
H3K27me1
RAD51
RPA1A
Input
H3K27ac
H3K4me3
ETS
18 S
5.8 S
25 S
Chr3:14195-14205kb
b
RAD51 ChIP
Col-0 Mock
atxr5 atxr6 Mock
ncRNA (n=630)
TE (n=1861)
H3K27me1 ChIP
Col-0 Untreated
atxr5 atxr6 Untreated
-2.0 TSS TES 2.0 kb
c
RPA1A ChIP
ncRNA (n=53)
TE (n=409)
H3K27me1 ChIP
d
Input for RAD51 ChIP
Overall n=16865
45S rDNA n=2
ncRNA (n=630)
TE (n=1861)
PCG n=9917
e
Input for RPA1A ChIP
Overall n=1675
45S rDNA n=2
ncRNA (n=53)
TE (n=409)
PCG n=514

Supplementary Fig. 10. ChIP signal of RAD51 and RPA1A in mock-treated Col-0 and *atxr5 atxr6*. a, IGV files of normalized ChIP signals (RPKM) of H3K9me2, H3K27me1, RAD51 (normalized with the reads of mitochondrial DNA), RPA1A (normalized with the reads of mitochondrial DNA), H3K27ac and H3K4me3 on a *45S rDNA* locus on chr3. H3K9me2 and H3K27me1 related data were mined from published data (GSE111814), H3K4me3 related data were mined from GSE166897, H3K27Ac related data were mined from GSE146126. Scales for the distinct loci were shown in left as solid lines. **b,** Distribution of normalized RAD51 ChIP-signal (normalized with the reads of mitochondrial DNA), and H3K27me1(RPKM) from Col-0 and *atxr5 atxr6* over RAD51-enriched different categories. H3K27me1 related data were mined from GSE111814. **c,** Distribution of normalized RPA1A ChIP-signal (normalized with the reads of mitochondrial DNA), and H3K27me1 (RPKM) from Col-0 and *atxr5 atxr6* over RPA1A enriched different categories. H3K27me1 related data were mined from GSE111814. **d,** Distribution of normalized input reads (RPKM) from Col-0 and *atxr5 atxr6* plants on different categories of RAD51 enriched regions. **e,** Distribution of normalized input reads from Col-0 and *atxr5 atxr6* plants on different categories of RPA1A enriched regions.

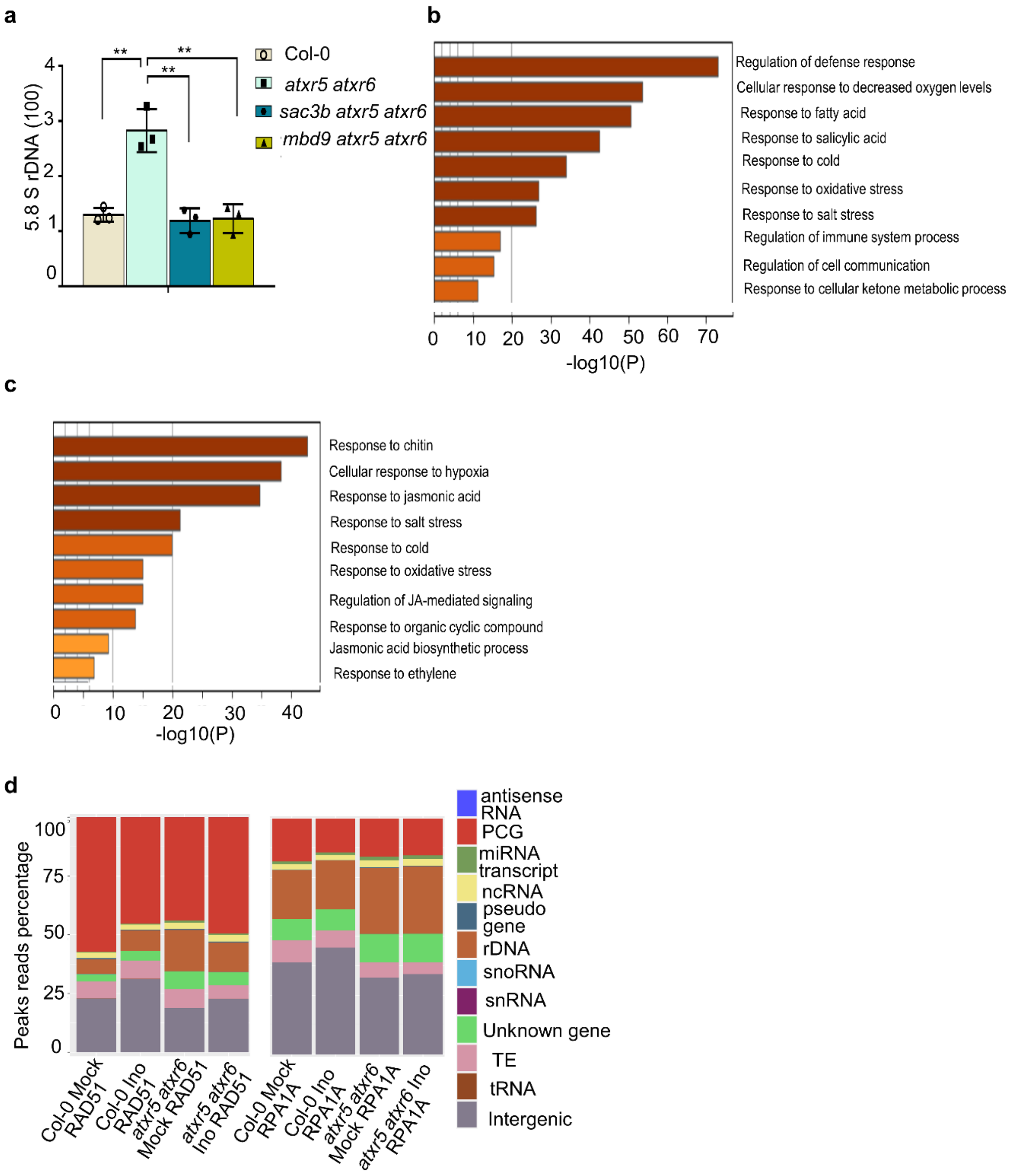

a
4
3
2
1
0
5.8 S rDNA (100)
**
**
**
Col-0
atxr5 atxr6
sac3b atxr5 atxr6
mbd9 atxr5 atxr6
b
Regulation of defense response
Cellular response to decreased oxygen levels
Response to fatty acid
Response to salicylic acid
Response to cold
Response to oxidative stress
Response to salt stress
Regulation of immune system process
Regulation of cell communication
Response to cellular ketone metabolic process
0 10 20 30 40 50 60 70
-log10(P)
c
Response to chitin
Cellular response to hypoxia
Response to jasmonic acid
Response to salt stress
Response to cold
Response to oxidative stress
Regulation of JA-mediated signaling
Response to organic cyclic compound
Jasmonic acid biosynthetic process
Response to ethylene
0 10 20 30 40
-log10(P)
d
Peaks reads percentage
100
75
50
25
0
Col-0 Mock RAD51
Col-0 Ino RAD51
atxr5 atxr6 Mock RAD51
atxr5 atxr6 Ino RAD51
Col-0 Mock RPA1A
Col-0 Ino RPA1A
atxr5 atxr6 Mock RPA1A
atxr5 atxr6 Ino RPA1A
antisense RNA
PCG
miRNA transcript
ncRNA
pseudo gene
rDNA
snoRNA
snRNA
Unknown gene
TE
tRNA
Intergenic

Supplementary Fig. 11. Gene Ontology (GO) analysis of RAD51 and RPA1A enriched PCGs. a, q-PCR shows the amount of rDNA in Col-0, *atxr5 atxr6*, *mbd9 atxr5 atxr6,* and *sac3b atxr5 atxr6*. The relative amount of rDNA was normalized against *UBQ10* (Unpaired two tailed student t-test, *, **, *** and ****, *P* < 0.05, 0.01, 0.001, and 0.0001, respectively). Each dot in the bar plot represents one replicate, the experiments were performed with 36 plants/replicate. Data are presented as mean ± SD (n = 3 biological replicates). **b,** GO analysis shows the enrichment of RAD51 enriched top 3000 genes filtered by *P*-value in different biological processes. **c,** GO analysis show the enrichment of RPA1A-enriched genes in different biological processes. **d,** Peak reads distributions of RAD51 and RPA1A ChIP-seq in various locus categories in Col-0 and *atxr5 atxr6*. The y axis represents the percentage of reads mapped to loci belonging to various categories. GO analysis in the **Supplementary Fig. 11 b**, **c** was done via Metascape[https://metascape.org]. Source data are provided in the Source Data File.

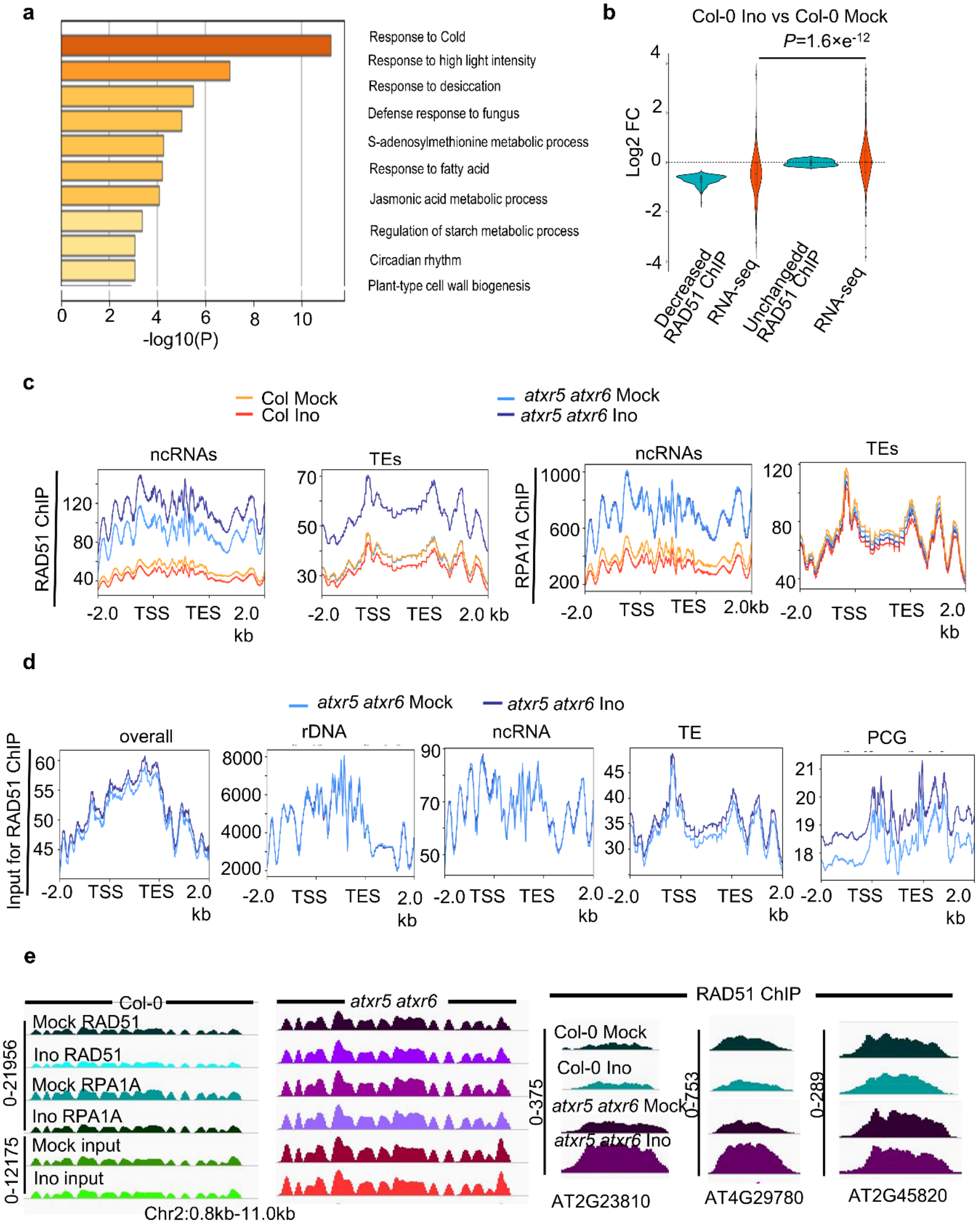
a
Response to Cold
Response to high light intensity
Response to desiccation
Defense response to fungus
S-adenosylmethionine metabolic process
Response to fatty acid
Jasmonic acid metabolic process
Regulation of starch metabolic process
Circadian rhythm
Plant-type cell wall biogenesis
0 2 4 6 8 10
-log10(P)
b
Col-0 Ino vs Col-0 Mock
P=1.6×e-12
Log2 FC
4 2 0 -2 -4
Decreased RAD51 ChIP
RNA-seq
Unchangedd RAD51 ChIP
RNA-seq
c
Col Mock
Col Ino
atxr5 atxr6 Mock
atxr5 atxr6 Ino
RAD51 ChIP
ncRNAs
TEs
RPA1A ChIP
ncRNAs
TEs
-2.0 TSS TES 2.0 kb
d
atxr5 atxr6 Mock
atxr5 atxr6 Ino
Input for RAD51 ChIP
overall
rDNA
ncRNA
TE
PCG
-2.0 TSS TES 2.0 kb
e
Col-0
atxr5 atxr6
Mock RAD51
Ino RAD51
Mock RPA1A
Ino RPA1A
Mock input
Ino input
0-21956
0-12175
Chr2:0.8kb-11.0kb
RAD51 ChIP
Col-0 Mock
Col-0 Ino
atxr5 atxr6 Mock
atxr5 atxr6 Ino
0-375
0-753
0-289
AT2G23810
AT4G29780
AT2G45820

Supplementary Fig. 12. The change of RAD51 and RP1A signal induced by viral infection in Col-0 and *atxr5 atxr6*. a, GO analysis of PCGs show the decreased RAD51 signal over the indicated pathways in Col-0 upon viral inoculation. Go analysis was done via Metascape[https://metascape.org]. **b,** Violin plot shows the transcript changes from the loci with reduced and unchanged RAD51 ChIP signal in viral-inoculated Col-0 vs Mock-treated Col-0. Horizontal lines in the bar plots display the 75th percentile, median, and 25th percentile, respectively. Whiskers represents the minimum and maximum values. *P* value is calculated by unpaired two-tailed Welch's approximate *t*-test. **c,** Distribution of normalized RAD51 and RPA1A ChIP-signal (normalized with the reads of mitochondrial DNA) from mock-treated and virus-inoculated Col-0 and *atxr5 atxr6* over RAD51 or RPA1A enriched different categories. **d,** Distribution of normalized input reads (RPKM) from mock-treated and virus-inoculated *atxr5 atxr6* on different categories of RAD51-enriched regions. **e,** IGV files show changes of RAD51 and RPA1A signal of an rDNA loci and indicated PCGs in mock-treated and virus-inoculated Col and *atxr5 atxr6* (normalized by RPKM). Scales for the distinct loci were shown in left as solid lines.

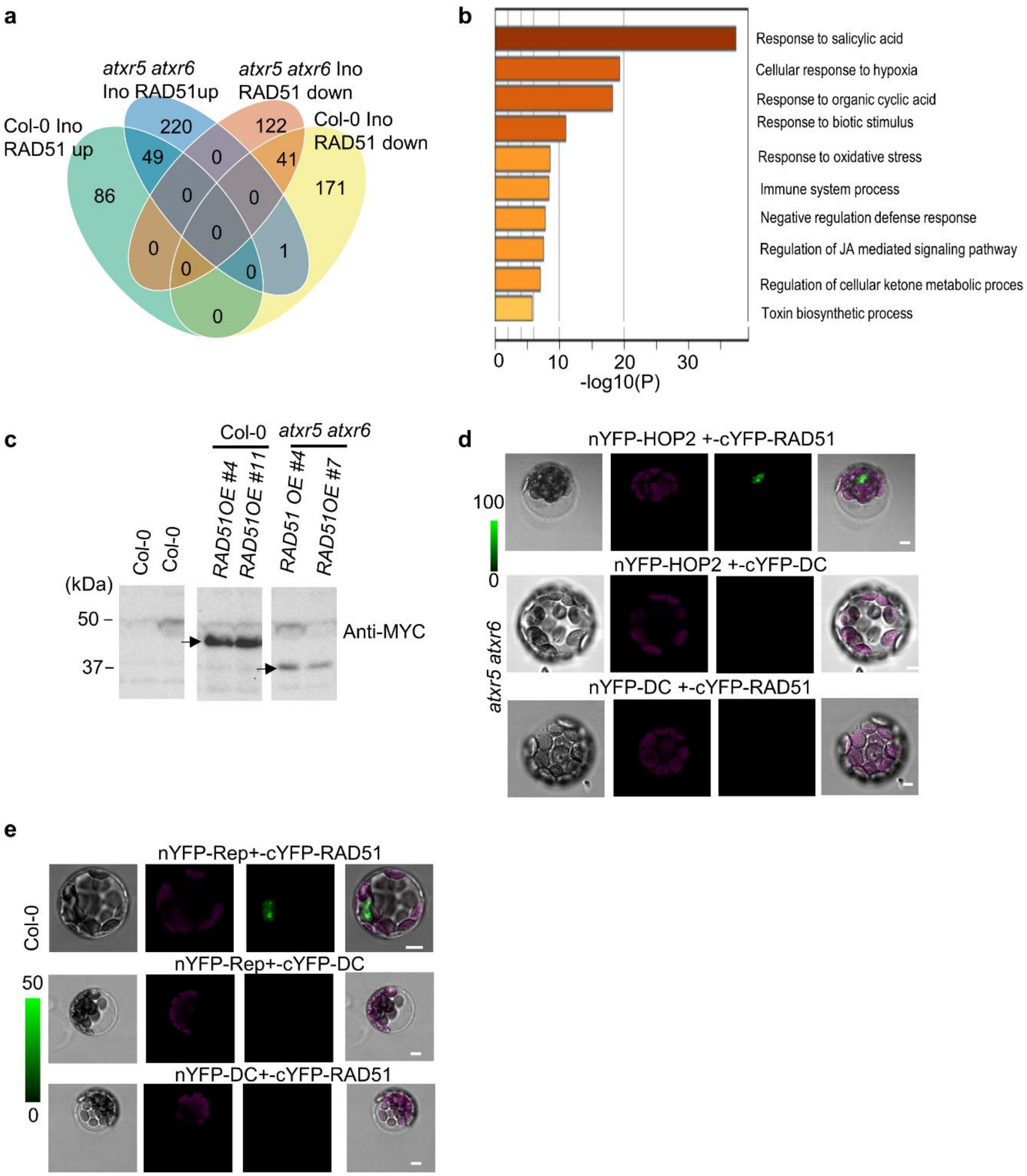
a
atxr5 atxr6 Ino RAD51up
atxr5 atxr6 Ino RAD51 down
Col-0 Ino RAD51 up
Col-0 Ino RAD51 down
220
122
49
0
41
86
171
0
0
0
0
1
0
0
0
b
Response to salicylic acid
Cellular response to hypoxia
Response to organic cyclic acid
Response to biotic stimulus
Response to oxidative stress
Immune system process
Negative regulation defense response
Regulation of JA mediated signaling pathway
Regulation of cellular ketone metabolic proces
Toxin biosynthetic process
0
10
20
30
-log10(P)
c
Col-0
atxr5 atxr6
Col-0
Col-0
RAD51OE #4
RAD51OE #11
RAD51 OE #4
RAD51OE #7
(kDa)
50
37
Anti-MYC
d
nYFP-HOP2 +-cYFP-RAD51
100
0
nYFP-HOP2 +-cYFP-DC
atxr5 atxr6
nYFP-DC +-cYFP-RAD51
e
nYFP-Rep+-cYFP-RAD51
Col-0
nYFP-Rep+-cYFP-DC
50
0
nYFP-DC+-cYFP-RAD51

Supplementary Fig. 13. Viral infection enhances the RAD51 signal over PCGs related to defense response. a, Venn graph shows change of RAD51 signal from enriched peaks over host genome in Col-0 and *atxr5 atxr6* upon virus inoculation. **b,** GO analysis of PCGs show the enhanced RAD51 signal in *atxr5 atxr6* upon viral infection. GO analysis was done via Metascape tool[76]. **c,** Western blots analysis of RAD51 transgenic lines in Col-0 and *atxr5 atxr6* backgrounds (head arrows indicate the Flag-4Myc-RAD51 in Col-0 or 6Myc-RAD51 in *atxr5 atxr6*). The experiments were repeated twice with similar results. **d,** Confocal images showed the interaction between HOP2 and RAD51 in protoplasts from the indicated backgrounds. nYFP-DC and cYFP-DC served as negative controls. Scale bars, 5 μm. **e,** Confocal images showed the interaction between Rep and RAD51 in protoplasts from the indicated backgrounds. nYFP-DC and cYFP-DC served as negative controls. Scale bars, 5 μm. Source data are provided in the Source Data File.

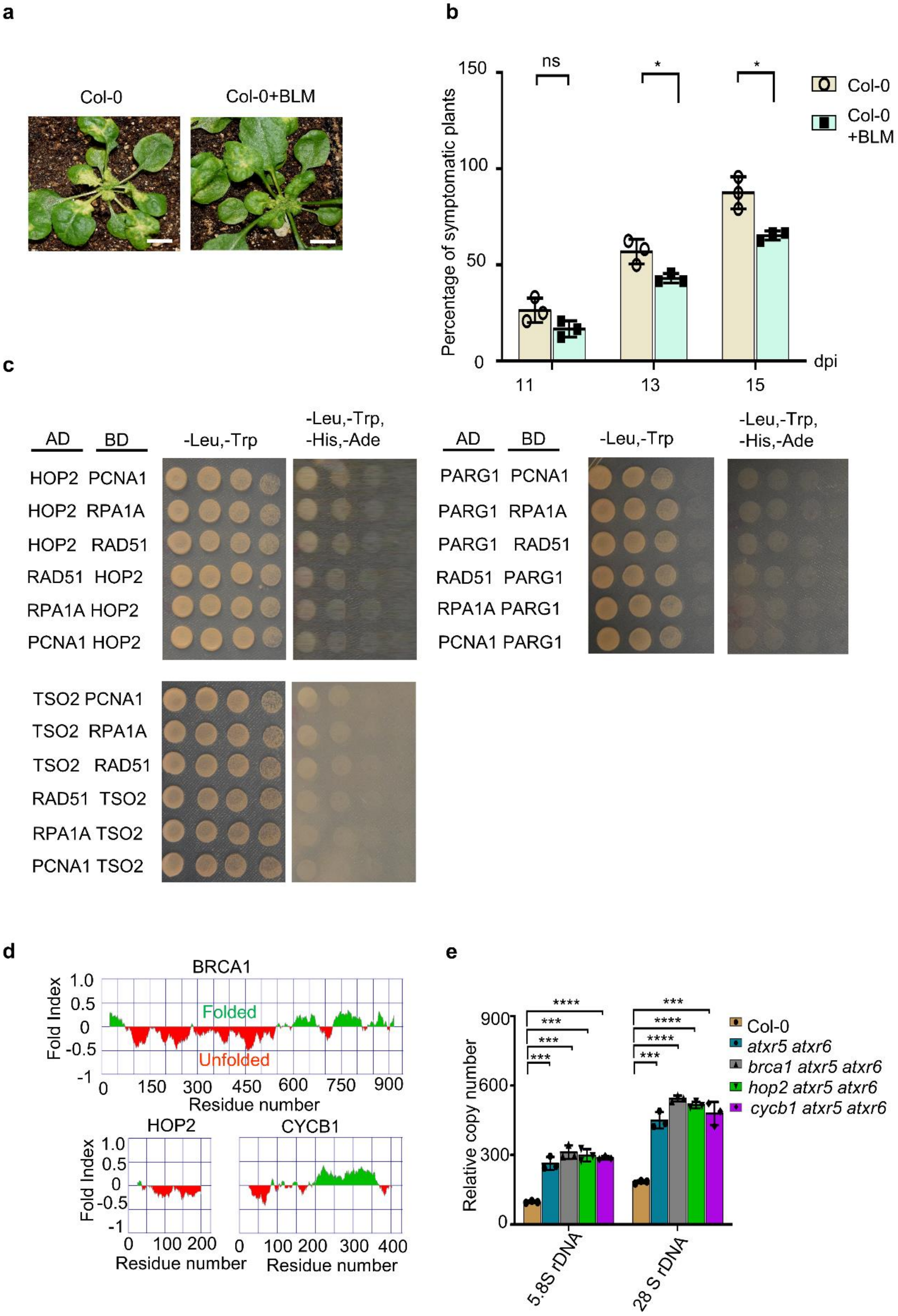
a
Col-0
Col-0+BLM
b
ns
*
*
150
100
50
0
Percentage of symptomatic plants
11
13
15
dpi
Col-0
Col-0 +BLM
c
AD BD
-Leu,-Trp
-Leu,-Trp, -His,-Ade
HOP2 PCNA1
HOP2 RPA1A
HOP2 RAD51
RAD51 HOP2
RPA1A HOP2
PCNA1 HOP2
PARG1 PCNA1
PARG1 RPA1A
PARG1 RAD51
RAD51 PARG1
RPA1A PARG1
PCNA1 PARG1
TSO2 PCNA1
TSO2 RPA1A
TSO2 RAD51
RAD51 TSO2
RPA1A TSO2
PCNA1 TSO2
d
BRCA1
Fold Index
Folded
Unfolded
Residue number
HOP2
CYCB1
Residue number
Residue number
e
****
***
***
***
***
****
****
***
900
600
300
0
Relative copy number
Col-0
atxr5 atxr6
brca1 atxr5 atxr6
hop2 atxr5 atxr6
cycb1 atxr5 atxr6
5.8S rDNA
28 S rDNA

Supplementary Fig. 14. Deletions of HOP2, BRCA1, and CYCB1 do not affect the viral DNA replication. a, Representative phenotypes of CaLCuV-inoculated plants in the indicated background and treatment. Photographs were taken at 15 dpi. Scale bars, 2 cm. **b,** Percentages of symptomatic plants induced by CaLCuV-inoculation in the indicated background and treatment at 11, 13, and 15 dpi. Each dot in the bar plot represents one replicate, experiments were performed with 24 plants/replicate. Data are presented as mean ± SD (n = 3 biological replicates). **c,** Y2H assays to test the interaction between RAD51, RPA1A and selected candidate factors. **d,** Folding prediction of HOP2, BRCA1 and CYCB1. **e,** q-PCR shows the amount of rDNA in Col-0, *atxr5 atxr6*, *brca1 atxr5 atxr6*, *hop2 atxr5 atxr6,* and *cycb1 atxr5 atxr6*. Each dot in the bar plot represents one replicate, experiments were performed with 32 plants/replicate. Data are presented as mean ± SD (n = 3 biological replicates). The relative amount of rDNA was normalized against *UBQ10*. (Unpaired two-tailed student t-test, *, **, *** and ****, *P* < 0.05, 0.01, 0.001, and 0.0001, respectively). Source data are provided in the Source Data File.

Genotype	DNA re-replication	TEs re-activation	Activation of DDR	Virus resistance
Col-0	No	No	No	No
atxr5 atxr6	Yes	Yes	Yes	Yes
brca1 atxr5 atxr6	Yes	Yes	No	No
mbd9 atxr5 atxr6	No	No	No	No
sac3b atxr5 atxr6	No	No	No	No

Supplementary Table 1 Summary of the performance of triple mutants regarding the heterochromatin amplification, TE re-activation, DDR, and viral susceptibility.

Gene ID	Go enriched pathway	Go enriched pathway
AT2G01440	DNA repair	Response to DNA damage
AT2G17930	DNA repair	Response to DNA damage
AT3G50880	DNA repair	Response to DNA damage
AT1G15940	DNA repair	Response to DNA damage
AT1G20750	DNA repair	Response to DNA damage
AT2G31320	DNA repair	Response to DNA damage
AT2G31970	DNA repair	Response to DNA damage
AT3G07800	DNA repair	Response to DNA damage
AT3G02680	DNA repair	Response to DNA damage
AT3G27060	DNA repair	Response to DNA damage
AT3G24170	DNA repair	Response to DNA damage
AT4G21070	DNA repair	Response to DNA damage
AT4G25120	DNA repair	Response to DNA damage
AT4G25540	DNA repair	Response to DNA damage
AT5G02820	DNA repair	Response to DNA damage
AT5G45400	DNA repair	Response to DNA damage
AT5G61460	DNA repair	Response to DNA damage
AT3G27630		Response to DNA damage
AT1G68790		Response to DNA damage

Supplementary Table 2 GO analysis of 19 DDR genes from Fig. 2b

Sample_name	mt DNA Ratio
*atxr5 atxr6*_Ino_rep1_Input	2.00%
*atxr5 atxr6*_Ino_rep2_Input	2.09%
*atxr5 atxr6*_Ino_rep1_RAD51	0.55%
*atxr5 atxr6*_Ino_rep1_RPA1A_	1.60%
*atxr5 atxr6*_Ino_rep2_RAD51_	0.56%
*atxr5 atxr6*_Ino_rep2_RPA1A_	1.53%
*atxr5 atxr6*_Mock_rep1_Input	2.11%
*atxr5 atxr6*_Mock_rep2_Input	2.03%
*atxr5 atxr6*_Mock_rep1_RAD51	0.89%
*atxr5 atxr6*_Mock_rep1_RPA1A	1.55%
*atxr5 atxr6*_Mock_rep2_RAD51	0.77%
*atxr5 atxr6*_Mock_rep2_RPA1A	1.52%
Col-0_Ino_rep1_Input	1.92%
Col-0_Ino_rep2_Input	1.95%
Col-0_Ino_rep1_RAD51	0.70%
Col-0_Ino_rep1_RPA1A	1.63%
Col-0_Ino_rep2_RAD51	0.80%
Col-0_Ino_rep2_RPA1A	1.48%
Col-0_Mock_rep1_Input	1.96%
Col-0_Mock_rep2_Input	1.96%
Col-0_Mock_rep1_RAD51	0.68%
Col-0_Mock_rep1_RPA1A	1.42%
Col-0_Mock_rep2_RAD51	0.74%
Col-0_Mock_rep2_RPA1A	1.46%

Supplementary Table 3 The ratios of mitochondria-mapped reads from ChIP-seq
