# Supplementary material for: H3.1K27me1 loss confers Arabidopsis resistance to Geminivirus by sequestering DNA repair proteins onto host genome
Source: Nat Commun. 2023 Nov 18;14:7484. doi: 10.1038/s41467-023-43311-1 (PMC10657422; doi:10.1038/s41467-023-43311-1)
Supplement: Supplementary file 3 — Description of Additional Supplementary Files [file 41467_2023_43311_MOESM3_ESM.pdf]

Description of Additional Supplementary Files:

Supplementary Data 1: Gene lists used in Fig. 2a and b. List of 365 transcripts selected from a total of 4800 DEGs based on the clustering in Fig. 2a and Fig. 2b.

Supplementary Data 2: Gene lists used in Fig. 2d. List of the genes from Protein-protein interaction (PPI) network in Fig. 2d.

Supplementary Data 3: Gene lists used in Fig.4h. List of the genes that showed decreased RAD51 signal in Mock-atxr5 atxr6 vs Mock-Col-0 in Fig. 4h

Supplementary Data 4: Gene lists used in Supplementary Fig. 13a. List of the genes that displayed changed RAD51ChIP signal in Col-0 and atxr5 atxr6 upon CalCuV inoculation in Supplementary Fig. 13a.

Supplementary Data 5: Lists of primers used in the study. Primers used for genotyping and q-PCR.
