# Supplementary material for: H3.1K27me1 loss confers Arabidopsis resistance to Geminivirus by sequestering DNA repair proteins onto host genome
Source: Nat Commun. 2023 Nov 18;14:7484. doi: 10.1038/s41467-023-43311-1 (PMC10657422; doi:10.1038/s41467-023-43311-1)
Supplement: Supplementary file 9 — Reporting Summary [file 41467_2023_43311_MOESM9_ESM.pdf]

nature portfolio

Corresponding author(s): Xiuren Zhang

Last updated by author(s): Oct 26, 2023

Reporting Summary

Nature Portfolio wishes to improve the reproducibility of the work that we publish. This form provides structure for consistency and transparency in reporting. For further information on Nature Portfolio policies, see our Editorial Policies and the Editorial Policy Checklist.

Statistics

For all statistical analyses, confirm that the following items are present in the figure legend, table legend, main text, or Methods section.

n/a	Confirmed	
☐	☒	The exact sample size (*n*) for each experimental group/condition, given as a discrete number and unit of measurement
☐	☒	A statement on whether measurements were taken from distinct samples or whether the same sample was measured repeatedly
☐	☒	The statistical test(s) used AND whether they are one- or two-sided<br>*Only common tests should be described solely by name; describe more complex techniques in the Methods section.*
☒	☐	A description of all covariates tested
☐	☒	A description of any assumptions or corrections, such as tests of normality and adjustment for multiple comparisons
☐	☒	A full description of the statistical parameters including central tendency (e.g. means) or other basic estimates (e.g. regression coefficient) AND variation (e.g. standard deviation) or associated estimates of uncertainty (e.g. confidence intervals)
☐	☒	For null hypothesis testing, the test statistic (e.g. *F*, *t*, *r*) with confidence intervals, effect sizes, degrees of freedom and *P* value noted<br>*Give P values as exact values whenever suitable.*
☒	☐	For Bayesian analysis, information on the choice of priors and Markov chain Monte Carlo settings
☒	☐	For hierarchical and complex designs, identification of the appropriate level for tests and full reporting of outcomes
☒	☐	Estimates of effect sizes (e.g. Cohen's *d*, Pearson's *r*), indicating how they were calculated

Our web collection on statistics for biologists contains articles on many of the points above.

Software and code

Policy information about availability of computer code

Data collection	Libraries for strand specific RNA sequencing were sequenced on the Novaseq system with pair-end 150 bp read length (Novogene). Libraries for ChIP-seq were sequenced on the Novaseq system with pair-end 150 bp read length (Novogene).
Data analysis	RNA-seq<br>Normalization and differential expressed analysis were calculated by DESeq2 (ver.3.15). The visualization for selected loci was shown in Intergrative Genomics Viewer (IGV). Heat-map clustering was performed based on Pearson distance correlation.<br>ChIP-seq<br>Adapter trimming and mapping processes were performed as previously described. The leftover reads later were mapped by bowtie2 (ver. 2.4.4) with perfect matches using Arabidopsis genome TAIR10 (http://www. arabidopsis.org/) as a reference genome. Reads uniquely mapped to genome and reads mapped to multiple locations in the genome were separately extracted by Samtools (ver. 1.15.1) for distinct downstream peak calling analyses. MACS2 (ver. 2.2.5) was used for ChIP-seq peak calling using default parameters, both narrowPeak and broadPeak were called for each sample. We used the reads mapping to genome which contain sequence mapped to multiple locations in the genome to perform the ChIP signal profile, peak number, peak reads percentage analysis and comparative analysis with DESeq2 (ver.3.15). All downstream statistical analyses and plotted graphs were generated by R (ver. 4.0.2) and ggplot2. All ChIP-seq profiles for RAD51, RPA1A, and H3K27me1 were drawn by deeptools (ver. 3.7.4) using the default parameters.

For manuscripts utilizing custom algorithms or software that are central to the research but not yet described in published literature, software must be made available to editors and reviewers. We strongly encourage code deposition in a community repository (e.g. GitHub). See the Nature Portfolio guidelines for submitting code & software for further information.

Data

Policy information about availability of data

All manuscripts must include a data availability statement. This statement should provide the following information, where applicable:

- Accession codes, unique identifiers, or web links for publicly available datasets
- A description of any restrictions on data availability
- For clinical datasets or third party data, please ensure that the statement adheres to our policy

RNA-seq related data were mined from GSE77735.H3K9me2 and H3K27me1 related data were mined from GSE111814. H3K27Ac related data were mined from GSE146126 and H3K4me3 related data were mined from GSE166897. RAD51 and RPA related data were mined from GSE143582. The data generated during this study has been deposited in GEO (GSE235158) and will be released once the manuscript is accepted. Source data are provided with this paper.

Research involving human participants, their data, or biological material

Policy information about studies with human participants or human data. See also policy information about sex, gender (identity/presentation), and sexual orientation and race, ethnicity and racism.

Reporting on sex and gender	N/A
Reporting on race, ethnicity, or other socially relevant groupings	N/A
Population characteristics	N/A
Recruitment	N/A
Ethics oversight	N/A

Note that full information on the approval of the study protocol must also be provided in the manuscript.

Field-specific reporting

Please select the one below that is the best fit for your research. If you are not sure, read the appropriate sections before making your selection.

☒ Life sciences ☐ Behavioural & social sciences ☐ Ecological, evolutionary & environmental sciences

For a reference copy of the document with all sections, see nature.com/documents/nr-reporting-summary-flat.pdf

Life sciences study design

All studies must disclose on these points even when the disclosure is negative.

Sample size	Samples sizes for replicates were listed in the figure legends. For majority experiments, 36 plants are considered as 1 biological replicate
Data exclusions	No data exclsuion in this study
Replication	Two replicates for ChIP-seq and RNA-seq. Three replicates for ChIP-qPCR. Three replicates for majority virus inoculation
Randomization	For majority experiments, plants in the indicated background were grown in one tray contains 36 or 32 plants.
Blinding	No blinding used

Reporting for specific materials, systems and methods

We require information from authors about some types of materials, experimental systems and methods used in many studies. Here, indicate whether each material, system or method listed is relevant to your study. If you are not sure if a list item applies to your research, read the appropriate section before selecting a response.

Materials & experimental systems

n/a	Involved in the study	
[ ]	[x]	Antibodies
[x]	[ ]	Eukaryotic cell lines
[x]	[ ]	Palaeontology and archaeology
[x]	[ ]	Animals and other organisms
[x]	[ ]	Clinical data
[x]	[ ]	Dual use research of concern
[ ]	[x]	Plants

Methods

n/a	Involved in the study	
[ ]	[x]	ChIP-seq
[ ]	[x]	Flow cytometry
[x]	[ ]	MRI-based neuroimaging

Antibodies

Antibodies used

Anti-RAD51 (PHYTOAB, PHY1804A)
Anti-RPA1A (PHYTOAB, PHY1813S)
Anti-Myc (Sigma, C3956)
Anti-HA HA (Sigma, H9658)
Anti-H3 (Agrisera, AS10 710)

Validation

Anti-RAD51 (PHYTOAB, PHY1804A) The antibody is validated at https://www.phytoab.com/rad51%20antibody
Anti-RPA1A (PHYTOAB, PHY1813S). The antibody is validated at https://www.phytoab.com/rpa1a%20antibody
Anti-Myc (Sigma, C3956) The antibody is validated at https://www.sigmaaldrich.com/US/en/product/sigma/c3956
Anti-HA HA (Sigma, H9658) The antibody is validated at https://www.sigmaaldrich.com/US/en/product/sigma/h9658
Anti-H3 (Agrisera, AS10 710) The antibody is validated at https://www.agrisera.com/en/artiklar/h3-histone-h3.html

Dual use research of concern

Policy information about dual use research of concern

Hazards

Could the accidental, deliberate or reckless misuse of agents or technologies generated in the work, or the application of information presented in the manuscript, pose a threat to:

No	Yes	
[x]	[ ]	Public health
[x]	[ ]	National security
[x]	[ ]	Crops and/or livestock
[x]	[ ]	Ecosystems
[x]	[ ]	Any other significant area

Experiments of concern

Does the work involve any of these experiments of concern:

No	Yes	
[x]	[ ]	Demonstrate how to render a vaccine ineffective
[x]	[ ]	Confer resistance to therapeutically useful antibiotics or antiviral agents
[x]	[ ]	Enhance the virulence of a pathogen or render a nonpathogen virulent
[x]	[ ]	Increase transmissibility of a pathogen
[x]	[ ]	Alter the host range of a pathogen
[x]	[ ]	Enable evasion of diagnostic/detection modalities
[x]	[ ]	Enable the weaponization of a biological agent or toxin
[x]	[ ]	Any other potentially harmful combination of experiments and agents

Plants

Seed stocks

Seeds of drm1 drm2 cmt3 (CS16384), and clf-28 (SALK_139371) were obtained from Arabidopsis biological center (ABRC). T-DNA insertion mutants of CYCB1 (SALK_200647), HOP2 (SALK_136002), CHR31 (SALK_204501), TK1A (SALK_097767), RAD51 (SAIL_873_C08), RPA1A (SALK_017580) were purchased from ABRC and genotyped by PCR. Col-0 was used as a wild type and atxr5 atxr6, atxr5, atxr6, mbd9, mbd9 atxr5 atxr6, sac3b, sac3b atxr5 atxr6, brca1, brca1 atxr5 atxr6, atm, atr, sog1, atm atxr5 atxr6, fas2, htr1, htr2, htr3, htr9, htr1 htr2 htr3 htr9, suvh4 suvh5 suvh6 were described previously

Novel plant genotypes

N/A

Authentication

N/A

ChIP-seq

Data deposition

☒ Confirm that both raw and final processed data have been deposited in a public database such as GEO.

☒ Confirm that you have deposited or provided access to graph files (e.g. BED files) for the called peaks.

Data access links
May remain private before publication.

https://www.ncbi.nlm.nih.gov/geo/query/acc.cgi?acc=GSE235158

Files in database submission

Col_Moc_51_1.gz
Col_Moc_51_2.gz
Col_Moc_put_1.gz
Col_Moc_put_2.gz
Col_if_51_1.gz
Col_if_51_2.gz
Col_if_put_1.gz
Col_if_put_2.gz
atxr56_Moc_51_1.gz
atxr56_Moc_51_2.gz
atxr56_Moc_put_1.gz
atxr56_Moc_put_2.gz
atxr56_if_51_1.gz
atxr56_if_51_2.gz
atxr56_if_put_1.gz
atxr56_if_put_2.gz
atxr56_if_RPA_1.gz
atxr56_if_RPA_2.gz
atxr56_Moc_RPA_1.gz
atxr56_Moc_RPA_2.gz
Col_if_RPA_1.gz
Col_if_RPA_2.gz
Col_Moc_RPA_1.gz
Col_Moc_RPA_2.gz
Col_Moc_1.gz
Col_Moc_2.gz
atxr56_Moc_1.gz
atxr56 _Moc_2.gz
brca1atxr56_Moc_1.gz
brca1atxr56 _Moc_2.gz
sac3batxr56_Moc_1.gz
sac3batxr56 _Moc_2.gz
mbd9atxr56_Moc_1.gz
mbd9atxr56 _Moc_2.gz
Col_Ino_1.gz
Col_Ino_2.gz
atxr56_Ino_1.gz
atxr56 _Ino_2.gz
brca1atxr56_Ino_1.gz
brca1atxr56 _Ino_2.gz
sac3batxr56_Ino_1.gz
sac3batxr56 _Ino_2.gz
mbd9atxr56_Ino_1.gz

mbd9atxr56 _Ino_2.gz

Genome browser session (e.g. UCSC)

Arabidopsis genome TAIR10 (http://www. arabidopsis.org/)

Methodology

Replicates

Two replicates for ChIP-seq and RNA-seq. Three replicates for ChIP-qPCR. Three replicates for majority virus inoculation

Sequencing depth

Category Total Reads Total Mapped Reads
Col_Moc_51_1 7,282,156 6,921,208
Col_Moc_51_2 8,188,694 7,844,174
Col_Moc_put_1 6,999,847 6,948,662
Col_Moc_put_2 7,107,035 7,055,614
Col_if_51_1 7,223,722 6,896,779
Col_if_51_2 9,631,908 9,051,212
Col_if_put_1 5,943,046 5,793,523
Col_if_put_2 6,555,811 6,371,348
atxr56_Moc_51_1 8,049,886 7,717,158
atxr56_Moc_51_2 10,791,493 10,255,667
atxr56_Moc_put_1 4,970,366 4,932,923
atxr56_Moc_put_2 6,107,482 6,062,370
atxr56_if_51_1 9,840,944 7,973,965
atxr56_if_51_2 13,029,399 11,316,106
atxr56_if_put_1 9,238,669 9,024,897
atxr56_if_put_2 8,497,066 8,311,045
atxr56_if_RPA_1 8,587,694 6,700,372
Category Uniq mapped reads
Col_Moc_1.gz 31400000
Col_Moc_2.gz 34000000
atxr56_Moc_1.gz 37700000
atxr56 _Moc_2.gz 55700000
brca1atxr56_Moc_1.gz 43600000
brca1atxr56 _Moc_2.gz 36000000
sac3batxr56_Moc_1.gz 43600000
sac3batxr56 _Moc_2.gz 36000000
mbd9atxr56_Moc_1.gz 52800000
mbd9atxr56 _Moc_2.gz 56500000
Col_Ino_1.gz 18100000
Col_Ino_2.gz 16800000
atxr56_Ino_1.gz 31800000
atxr56 _Ino_2.gz 22600000
brca1atxr56_Ino_1.gz 31400000
brca1atxr56 _Ino_2.gz 31600000
sac3batxr56_Ino_1.gz 27000000
sac3batxr56 _Ino_2.gz 13800000
mbd9atxr56_Ino_1.gz 32500000
mbd9atxr56 _Ino_2.gz 38100000

atxr56_if_RPA_2 6,415,557 5,005,402
atxr56_Moc_RPA_1 9,191,184 7,229,166
atxr56_Moc_RPA_2 5,741,163 4,346,598
Col_if_RPA_1 8,458,950 6,639,682
Col_if_RPA_2 6,669,760 5,078,397
Col_Moc_RPA_1 6,549,419 5,199,328
Col_Moc_RPA_2 6,294,787 4,876,285

Antibodies

Anti-RAD51 (PHYTOAB, PHY1804A)
Anti-RPA1A (PHYTOAB, PHY1813S)

Peak calling parameters

MACS2 (ver. 2.2.5) was used for ChIP-seq peak calling using default parameters, both narrowPeak and broadPeak were called for each sample.

Data quality

We only keep the peak with P-value<0.05

Software

Adapter trimming and mapping processes were performed as previously described. The leftover reads later were mapped by bowtie2 (ver. 2.4.4) with perfect matches using Arabidopsis genome TAIR10 (http://www. arabidopsis.org/) as a reference genome. Reads uniquely mapped to genome and reads mapped to multiple locations in the genome were separately extracted by Samtools (ver. 1.15.1) for distinct downstream peak calling analyses. MACS2 (ver. 2.2.5) was used for ChIP-seq peak calling using default

parameters, both narrowPeak and broadPeak were called for each sample. All downstream statistical analyses and plotted graphs were generated by R (ver. 4.0.2) and ggplot2. All ChIP-seq profiles for RAD51, RPA1A, and H3K27me1 were drawn by deeptools (ver. 3.7.4) using the default parameters.

Flow Cytometry

Plots

Confirm that:

- [x] The axis labels state the marker and fluorochrome used (e.g. CD4-FITC).
- [x] The axis scales are clearly visible. Include numbers along axes only for bottom left plot of group (a 'group' is an analysis of identical markers).
- [x] All plots are contour plots with outliers or pseudocolor plots.
- [x] A numerical value for number of cells or percentage (with statistics) is provided.

Methodology

Sample preparation	Arabidopsis
Instrument	BD Fortessa X-20
Software	Flow Jo
Cell population abundance	10000
Gating strategy	Texas Red signal>400

- [x] Tick this box to confirm that a figure exemplifying the gating strategy is provided in the Supplementary Information.
